# Gut microbiomes of agropastoral children from the Adadle region of Ethiopia reflect their unique dietary habits

**DOI:** 10.1038/s41598-023-47748-8

**Published:** 2023-12-01

**Authors:** Simon Yersin, Julian R. Garneau, Pierre H. H. Schneeberger, Kadra Ali Osman, Colin Ivano Cercamondi, Abdifatah Muktar Muhummed, Rea Tschopp, Jakob Zinsstag, Pascale Vonaesch

**Affiliations:** 1 Department of Fundamental Microbiology, University of Lausanne, 1015 Lausanne, Switzerland; 2 Helminth Drug Development Unit, Swiss Tropical and Public Health Institute, Kreuzstrasse 2, 4123 Allschwil, Switzerland; 3 University of Basel, Petersplatz 1, 4001 Basel, Switzerland; 4 Jigjiga University, Jigjiga, Ethiopia; 5 Department of Health Sciences and Technology, ETHZ, Rämistrasse 101, 8092 Zurich, Switzerland; 6 Human and Animal Health Unit, Swiss Tropical and Public Health Institute, Kreuzstrasse 2, 4123 Allschwil, Switzerland; 7 Armauer Hansen Research Institute, Jimma Road, 1005 Addis Ababa, Ethiopia

**Keywords:** Microbiology, Gastroenterology

## Abstract

The composition and function of the intestinal microbiota are major determinants of human health and are strongly influenced by diet, antibiotic treatment, lifestyle and geography. Nevertheless, we currently have only little data on microbiomes of non-westernized communities. We assess the stool microbiota composition in 59 children aged 2–5 years from the Adadle district of Ethiopia, Somali Regional State. Here, milk and starch-rich food are predominant components of the local diet, where the inhabitants live a remote, traditional agropastoral lifestyle. Microbiota composition, function and the resistome were characterized by both 16S rRNA gene amplicon and shotgun metagenomic sequencing and compared to 1471 publicly available datasets from children living in traditional, transitional, and industrial communities with different subsistence strategies. Samples from the Adadle district are low in *Bacteroidaceae,* and *Prevotellaceae,* the main bacterial representatives in the feces of children living in industrialized and non-industrialized communities, respectively. In contrast, they had a higher relative abundance in *Streptococcaceae*,* Bifidobacteriaceae* and *Erysipelatoclostridiaceae*. Further, genes involved in degradation pathways of lactose, d-galactose and simple carbohydrates were enriched. Overall, our study revealed a unique composition of the fecal microbiota of these agropastoral children, highlighting the need to further characterize the fecal bacterial composition of human populations living different lifestyles.

## Introduction

The human gastro-intestinal tract microbiota plays a crucial role in immunity, brain development, metabolism and general health of human beings^1–4^. For the last two decades, the composition and function of the microbiome has been an area of intense and dynamic research facilitated by the advancement in sequencing methods and data analysis tools^5^. However, despite large-scale efforts in the characterization of the intestinal microbiota, many unknowns remain in our understanding of the colonization of our intestinal tract by microorganisms, their functionalities and their associations with non-communicable diseases^4,6,7^.

Factors, such as birth-mode, breast-feeding, diet, antibiotic treatment, diseases, and proximity with animals, have been shown to strongly influence the intestinal microbiota and vary widely among populations^8–11^. Such factors have led to significant variations in the composition of what is considered a “healthy microbiome”. The definition of a eubiotic community is crucial to develop microbiota-targeted interventions. Nevertheless, societies that live traditional lifestyles and communities currently undergoing a transition towards industrialization and urbanization remain understudied in comparison to populations from industrialized northern-American and European countries^12,13^. It is therefore crucial to better characterize the composition and function of the microbiome in diverse communities across the globe.

In recent years, studies on the intestinal microbiota of hunter-gatherer communities such as the Hadza from Tanzania or the Matses from Peru and Brazil, as well as other traditional populations such as agriculturalists from Malawi or Venezuela, showed an enrichment in members of the *Prevotellaceae, Spirochaetaceae* and *Succinivibrionacea*^14–17^. In contrast, the intestinal microbiota in subjects from industrialized societies has been associated with increased relative abundance of *Bacteroidaceae* and *Akkermensiaceae*^18–20^. The terms VANISH (volatile and/or associated negatively with industrialized societies of humans) and BloSSUM (bloom or selected in societies of urbanization/modernization) have been proposed to describe these taxa shared between populations with similar lifestyles^18^. While VANISH taxa are associated with a characteristic high-fiber diet of traditional communities, BloSSUM taxa correlate with the higher consumption of animal fat and protein in industrialized societies^18,21,22^.

Although mostly reported in adults, lifestyle has an equally important role in shaping the fecal microbiota composition in children^23,24^. During the first two years of life, the maturation of the intestinal microbiota is strongly influenced by factors including birth mode, breastfeeding, and diet^8,25^. Children’s gut microbiota continues to develop during childhood to stabilize towards an adult-like phylogenetic distribution later in life^26^. Growing evidences suggest that compositional alterations during this dynamic maturation and developmental period might have long-lasting effects on the health of an individual^8^.

In light of the important contribution of lifestyle and diet on the intestinal microbial community, the intestinal microbiota composition and microbial functional potential need to be studied and characterized in populations from across the globe with differing subsistence strategies, lifestyles and dietary preferences. Here, we assessed the intestinal microbiota in agropastoral children from the Adadle *woreda* (district) in the Somali regional state of Ethiopia. We used both16S rRNA gene amplicon as well as whole-genome shotgun metagenomic sequencing to compare these children to other children living in geographically distant sites and living different lifestyles. Due to their unique way of life and their specific diet, we hypothesized that these agropastoral children harbor a distinct microbiome profile compared to children living any other traditional lifestyle.

This study is part of the Jigjiga University One Health initiative (JOHI), aiming at the improvement of health and livelihoods of mobile pastoralists and their animals in the Somali Region of Ethiopia. It primarily aimed at assessing the nutritional status and health care of children^27,28^. In parallel, the status of antimicrobial resistance and the health status of animals are assessed, aiming towards an integrated surveillance-response system for human and animal health^29^.

## Results

### Description of study population

**Figure 1:**
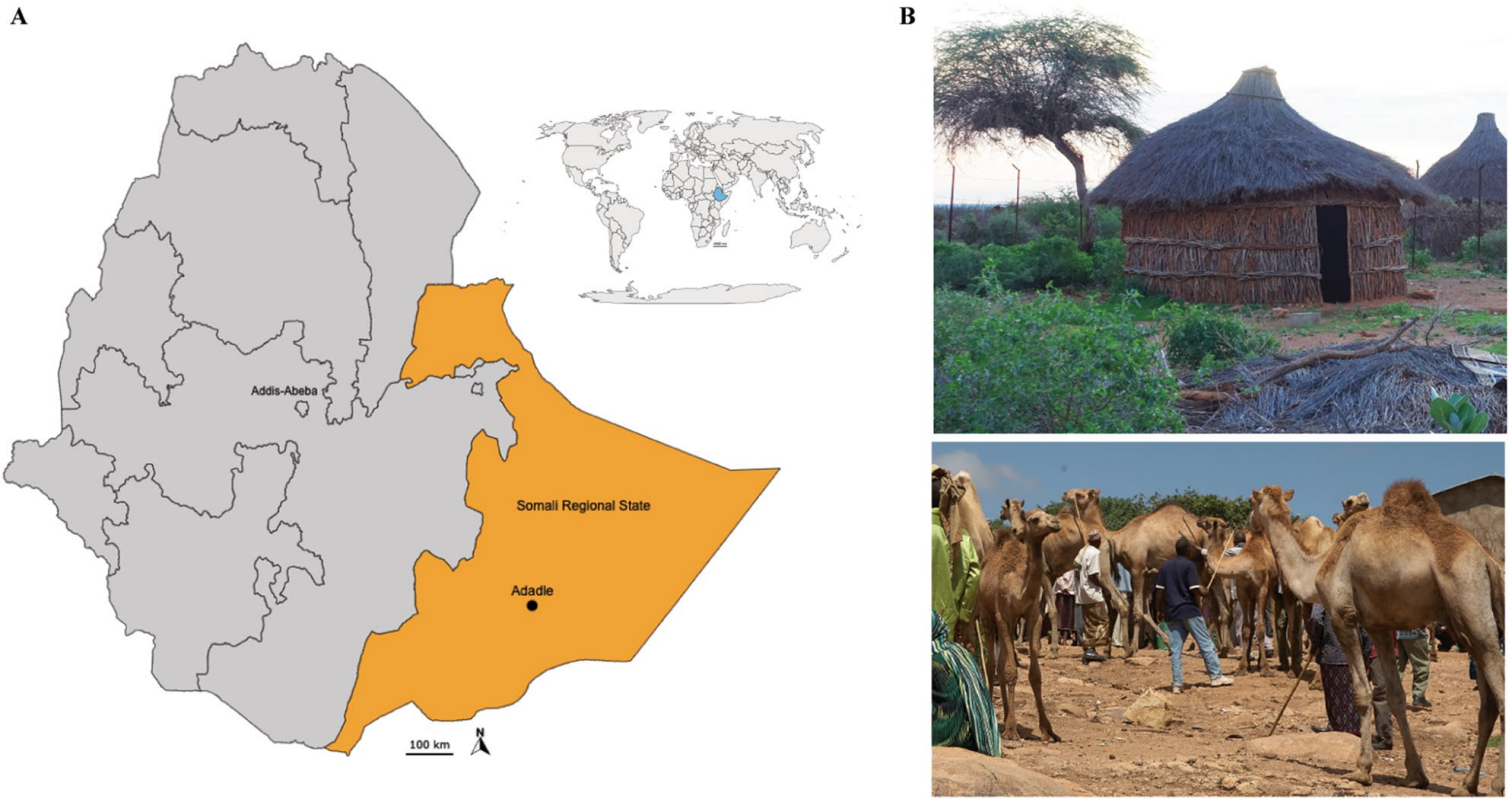
Sampling location and habitats of the studied agropastoral population. (**A**) Map of Ethiopia with the Somali Regional State highlighted in orange and Adadle *woreda* pinpointed. Upper right: map of the African continent with Ethiopia highlighted in blue. The maps were generated with GADM data (gadm.org, v4.0.4) and the magrit application (magrit.cnrs.fr, v0.8.14). (**B**) Habitats of the population. Top picture, Adadle *woreda*, Ethiopia. Bottom picture, camel market in Ethiopia (Photos courtesy of Pascale Vonaesch).

The Ethiopian population studied were agropastoralists from the Adadle *woreda* (district) in the Shabelle zone of the Somali Regional State. This region is mostly inhabited by pastoral and agropastoral communities that rely mainly on animals for food and livelihood (Fig. 1). This study included feces from children aged 2–5 years, living in traditional agropastoral communities in the Adadle *woreda*. Samples were collected in the context of a previous cross-sectional study on parasitic infection and micronutrient status conducted in this region in the dry season between July and September 2016^27^. Overall, 54 children were included in the final analysis using the first primer set (V4 region 501-508/701-712), 13 in the study using the second primer set (V4 region 515/806) and 15 children using shotgun metagenomic sequencing. Of the 54 children (primer set 1), 41% (22/54) were girls and 59% (32/54) were boys. Children were between 2 and 5 years old with the median age being 4 years of age (Table 1). In the 24-h dietary recall (Table 1), the main staple food consumed by the children included whole wheat (20% of the children) or wheat flour (15%), maize (29%), rice (19%), sorghum (4%) and potato (2%). Only few children were reported as having consumed tomato (15%) and onions (13%) but none had other vegetables, fruits, meat or fish. Additionally, 44 out of 54 children (82%) consumed animal milk (from camels, goats, sheep or cows) or tea with milk in the 24 h before sampling^27^. The metadata for primer set 1, 2 and shotgun metagenomic sequencing groups are shown in Table 1.

**Table 1:** Description of the study population.

Dataset	Primer set 1	Primer set 2	Shotgun metagenomic
N	54	13	15
Sequencing method	16S rRNA gene amplicon	16S rRNA gene amplicon	Shotgun metagenomic
Sex			
Female	41% (22/54)	31% (4/13)	47% (7/15)
Male	59% (32/54)	69% (9/13)	53% (8/15)
Age			
Median	4 years old	4 years old	4 years old
2–3 years	15% (8/54)	0% (0/13)	0% (0/15)
3–4 years	31% (17/54)	31% (4/13)	33% (5/15)
4–5 years	54% (29/54)	69% (9/13)	67% (10/15)
Food consumption			
Whole wheat	20% (11/54)	23% (3/13)	13% (2/15%)
Wheat flour	15% (8/54)	8% (1/13)	0% (0/15)
Maize	28% (15/54)	15% (2/13)	40% (6/15)
Rice	19% (10/54)	8% (1/13)	0% (0/15)
Sorghum	4% (2/54)	0% (0/13)	0% (0/15)
Potato	2% (1/54)	8% (1/13)	0% (0/15)
Tomato	15% (8/54)	8% (1/13)	27% (4/15)
Onions	13% (7/54)	8% (1/13)	7% (1/15)
Animal milk	82% (44/54)	77% (10/13)	100% (15/15)

### Composition of the fecal microbiota of children from the Adadle region, Ethiopia

Using primer set 1, we generated a total of 3,832,363 reads and an average of 70,970 ± 34,438 reads per subject. Negative control samples had an average of 173 ± 40 reads, ruling out any potential contamination. Out of the 1490 identified ASVs, 1294 were assigned to *Bacteria* or *Archaea* and were retained to explore the composition of the fecal microbiota of these children (Supplementary data 3, 4). In the 54 stool samples, 125 bacterial families from 21 different phyla were detected (Supplementary data 6), without applying any prevalence filter. After filtering at 10% prevalence, 12 phyla and 69 bacterial families with low prevalence were removed in the dataset. Filtering did not influence any conclusions from downstream analysis (Supplementary Fig. S1).

**Figure 2:**
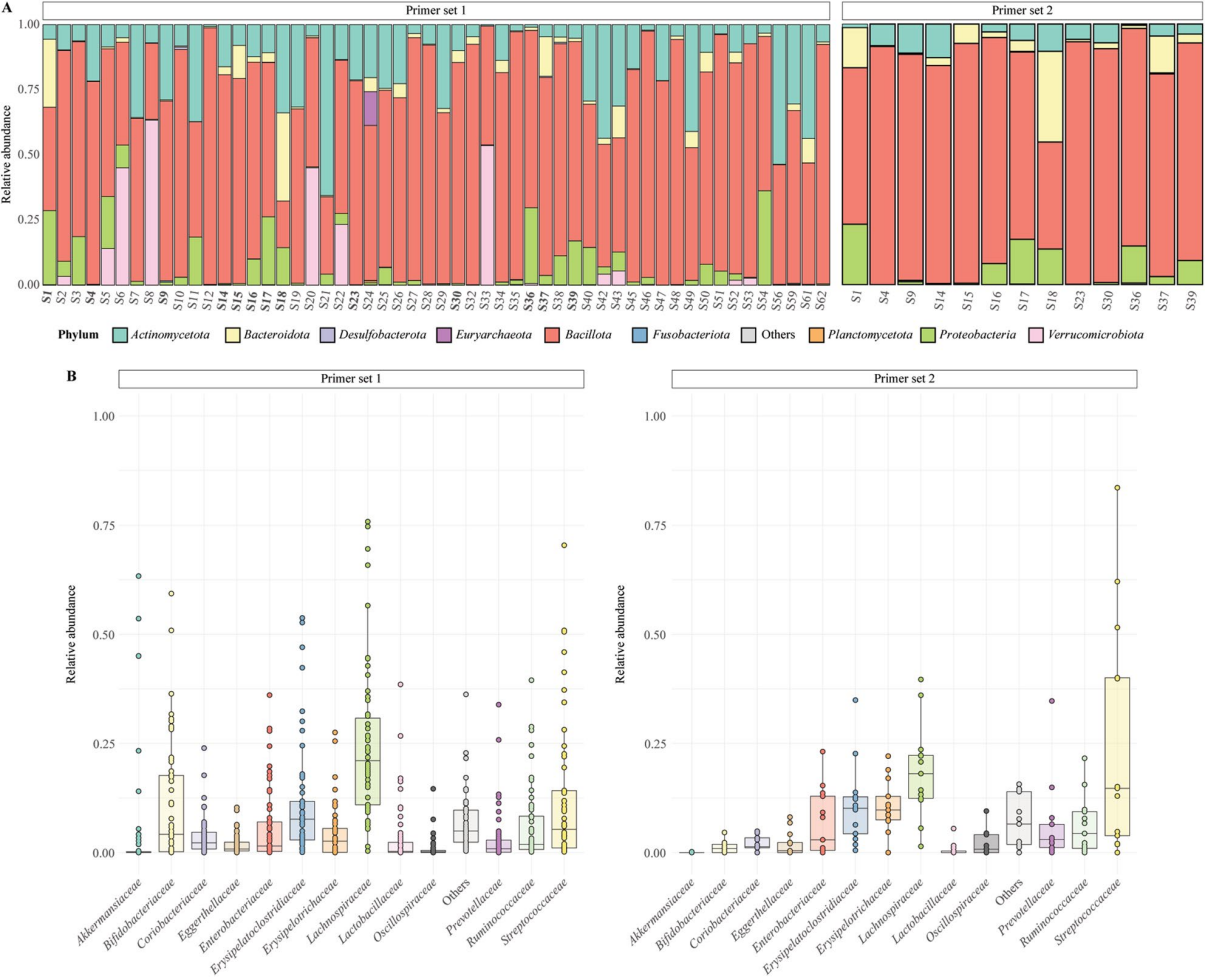
Composition of the fecal microbiota of children living in the Adadle region. Primer set 1 is targeting the V4 region 501–508/701–712, N = 54. Primer set 2 is targeting the V4 region 515/806, N = 13. (**A**) Relative abundance of the most abundant phyla for samples from the Adadle *woreda*. Less abundant phyla are grouped in the Others category. Samples in common in both datasets are highlighted in bold in the primer set 1 plot. (**B**) Box plot of the relative abundance of the most abundant bacterial families for samples from the Adadle *woreda*. The less abundant families are grouped in the Others category. Primer sets’ relative abundance and prevalence are compared using Wilcoxon rank test at a significance threshold of 0.05 with Bonferroni correction for multiple comparisons.

The samples were low in relative abundance of the phylum *Bacteroidota* (formerly known as *Bacteroidetes*, relative abundance: 3.5 ± 6.3%, prevalence: 54/54), including mostly the *Prevotellaceae* family (relative abundance: 3.1%, prevalence: 52/54) (Fig. 2). There was a high percentage of *Actinomycetota* (formerly known as *Actinobacteria*, relative abundance: 16.8 ± 15.6%, prevalence: 54/54), especially of *Bifidobacteriaceae* (relative abundance: 10.8%, prevalence: 54/54) (Fig. 2). The samples were high in both prevalence and relative abundance of *Erysipelatoclostridiaceae* (relative abundance: 11.3%, prevalence: 54/54), *Streptococcaceae* (relative abundance: 12.3%, prevalence: 54/54), *Erysipelotrichaceae* (relative abundance: 4.3%*,* prevalence: 52/54) and *Lactobacillaceae* (relative abundance: 3.3%, prevalence: 54/54). Most strikingly, the level of *Akkermansiaceae* (relative abundance: 4.9%, prevalence: 44/54) were high in several samples (Fig. 2, Supplementary data 6).

Using primer set 2, with the 13 samples that passed quality control, we generated 98,908 reads with an average of 7608 ± 2421 reads per samples and 1,197 identified ASVs assigned to *Bacteria* (Supplementary data 3, 5). Negative control samples for primer set 2 failed the DADA2 pipeline due to low read count, ruling out potential contamination. When assessing for the composition of the microbiota in the reduced dataset shared between both primer set, we noted that the composition of the samples was largely similar in terms of the main taxa recovered as well as their relative abundance (Fig. 2B, Supplementary Fig. S2, Supplementary data 6). A notable exception was the *Akkermansiaceae* bacterial family, whose prevalence was significantly lower (*p* value = 0.041) in the 515F/806R samples (2/13) compared to the primer set 2 dataset (11/13) (Fig. 2B, Supplementary data 6). Overall, these observations showed a commonly shared microbiome in the agropastoral children dominated by *Bacillota* (formerly known as *Firmicutes*) and *Actinomycetota* and low relative abundance of different members of *Bacteroidota*.

### Fecal samples from agropastoral children from the Adadle region are distinct compared to children from other geographic locations

**Figure 3:**
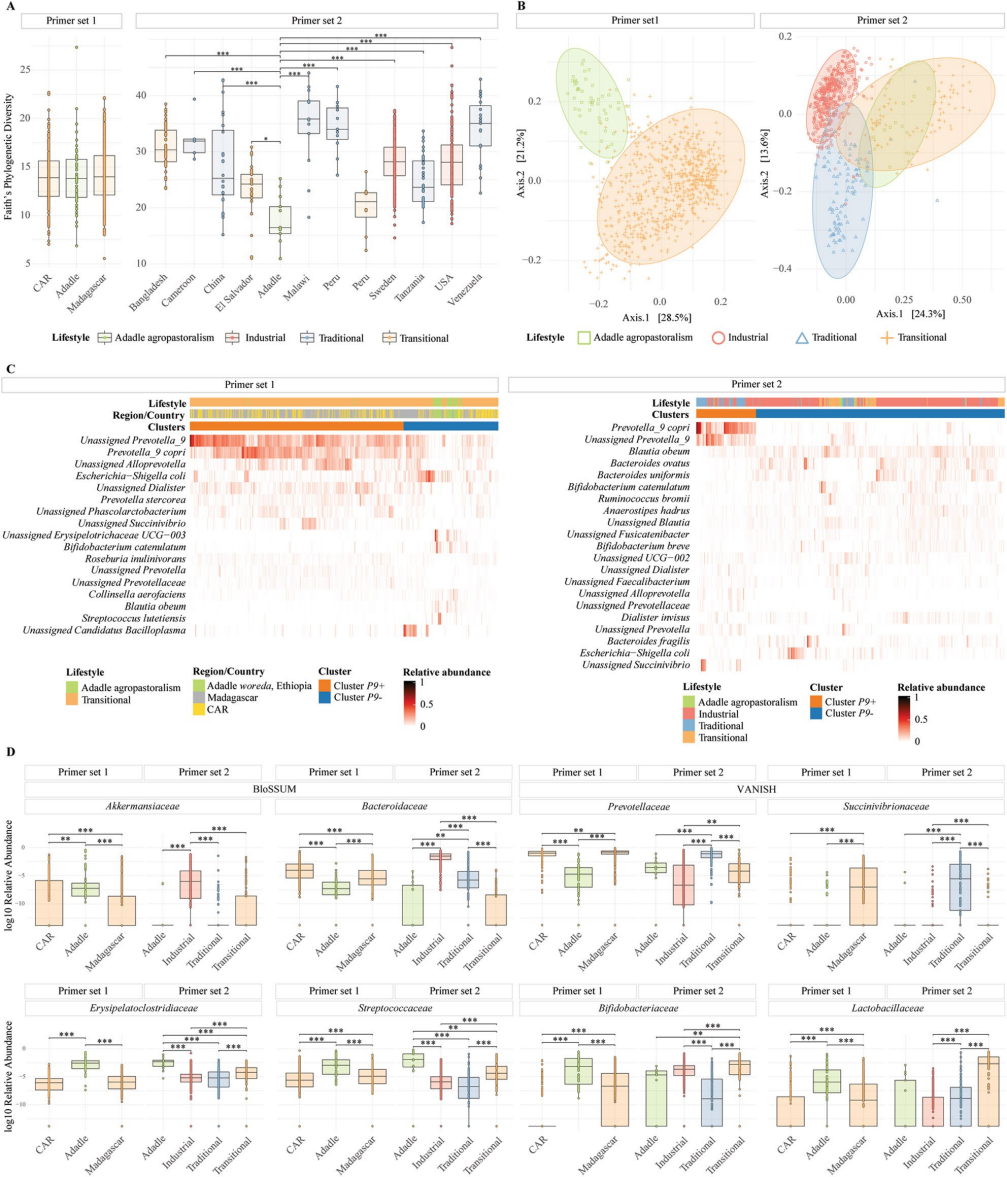
Children’s fecal microbiota composition from the Adadle *woreda* compared with children living on other subsistence strategies. Primer set 1 is targeting the V4 region 501–508/701–712, N = 759. Primer set 2 is targeting the V4 region 515/806, N = 692. (**A**) Alpha diversity measure as Faith's phylogenetic diversity at species level. Pairwise comparisons done using Wilcoxon rank test with Bonferroni correction for multiple comparisons (*p < 0.05; **p < 0.01; ***p < 0.001). (**B**) First and second coordinates of dimension reduction for WeightedUniFrac distance with the values indicating the amount of total variability explained by the coordinates. All pairwise comparisons were significant using PERMANOVA at a significance threshold of 0.05 using Benjamini–Hochberg correction for multiple comparisons. (**C**) Heatmap of the most abundant genera with significantly different relative abundance between the two clusters (P9+ and P9−). Relative abundance difference significance tested with Wilcoxon rank test at a significance threshold of 0.05 with Bonferroni correction for multiple comparisons and samples distribution tested using χ^2^-test at significance threshold of 0.05. (**D**) Boxplot of the log10 of the relative abundance of enriched or depleted taxa in the different communities. BloSSUM: Bloom or selected in societies of urbanization/modernization. VANISH: Volatile and/or associated negatively with industrialized societies of humans. Relative abundance test using Wilcoxon rank test at a significance threshold of 0.05 with Bonferroni correction for multiple comparisons (*p < 0.05; **p < 0.01; ***p < 0.001).

To test whether the intestinal microbiota of the children from the Adadle *woreda* is different from other traditional communities, we compared the microbiota composition between these children and data from previously published studies around the globe (Table 2). We first explored the species diversity within communities using Faith’s phylogenetic diversity (PD) and found that agropastoral children from the Adadle *woreda* have a similar species diversity than children from Madagascar and Central African Republic (CAR) (Fig. 3A). Using the primer set 2, we found that samples from the Adadle *woreda* have significantly lower phylogenetic diversity than children from other countries, except for children coming from the transitional population of Lima, Peru (Fig. 3A). Moreover, we found the same PD results on both primer set when applying a 0.25% filter on the taxa abundance (Supplementary Fig. S3A, C) as well as when rarefying multiple times and calculating the mean PD (Supplementary Fig. S3E–F). Overall, these results suggest that children from the Adadle *woreda* have a lower species diversity than children coming from traditional and industrial communities yet remains comparable to children from transitional populations from Africa and Peru.

Next, we applied Principal Coordinate Analysis (PCoA) of WeightedUniFrac (WUF) distance at the species level to assess for overall taxonomic composition of the samples. The ordination on the first, second and third components showed that samples from the Adadle *woreda* formed a clearly separated cluster compared to samples from Madagascar and CAR in the primer set 1 dataset (PERMANOVA *p *value < 0.005) (Axes 1 & 2: Fig. 3B, Axes 1 & 3, Axes 2 & 3: Supplementary Fig. S4). The ordination of the primer set 2 dataset showed that samples from the Adadle *woreda* clustered away from samples coming from industrial and traditional populations (PERMANOVA *p *value < 0.005). Further, even though samples from the Adadle *woreda* clustered more closely to samples from transitional communities, their microbiota composition was still significantly different (PERMANOVA *p *value < 0.005) (Fig. 3B). The same trend was confirmed using the Bray–Curtis, Jaccard and Generalized UniFrac distance metrics (Supplementary Figs. S4, S5). Moreover, we observed the same clustering of samples when applying a 0.25% abundance filter instead of removing singletons and using the Generalized UniFrac distance (Supplementary Figs. S3B, D).

Last, we used the Euclidean distance and the Ward’s linkage method for hierarchical clustering. We identified two clusters (*P9*+ and *P9−*), with the relative abundance of *Prevotella_9_copri* (Primer set 1 *p* value = 3.38e^−68^, Primer set 2 *p* value = 2.07e^−56^) and *Unassigned_Prevotella_9* (Primer set 1 *p* value = 8.46e^−60^, Primer set 2 *p* value = 1.84e^−65^) being the most significantly different between the two clusters (Wilcoxon rank test with Bonferroni correction for multiple comparisons) and the main driver separating the two clusters. In primer set 1 dataset, 50 out of the 54 samples and, in primer set 2 dataset, 11 out of 13 samples from the Adadle *woreda* clustered in *P9-*. Samples from Madagascar (328/431) and CAR (194/274) mostly clustered in *P9*+ (Fig. 3C)*.* While the samples’ cluster repartition between Madagascar and CAR was not significantly different (χ^2^ test *p* value > *0.05*), the repartition of samples from the Adadle *woreda* significantly differs from the two African countries (χ^2^ test *p* value < *0.05*) (Fig. 3C). Additionally, samples from industrial (422/484) and transitional (86/88) communities clustered mostly in *P9−* similar to samples from the Adadle *woreda* (χ^2^ test *p* value > 0.05) (Fig. 3C). Finally, 68 out of the 107 samples from traditional population clustered in *P9*+ with a repartition significantly different from samples from the Adadle *woreda* (χ^2^ test *p* value < 0.05). More specifically, most samples from Cameroon, China, Peru, and Tanzania clustered in *P9*+ and most samples from Malawi and Venezuela clustered in *P9−* (Fig. 3C). Thus, in conclusion, samples from the Adadle *woreda* cluster more closely to samples from transitional communities than with samples from populations adopting a traditional lifestyle.

### Analysis of enriched and depleted species in different communities

We further compared the relative abundance of specific bacterial families to assess for the compositional differences between samples from communities adopting different lifestyles. Samples from industrialized countries had high relative abundance of *Akkermansiaceae* and *Bacteroidaceae* (BloSSUM taxa) (Fig. 3D) whereas samples from traditional populations were high in the relative abundance of *Prevotellaceae* and *Succinivibrionaceae* (VANISH taxa) (Fig. 3D). Children from the Adadle region were found to have a significantly lower relative abundance of both BloSSUM and VANISH taxa compared to children from industrialized countries and traditional communities, respectively (Fig. 3D, *p* value < 0.05). Additionally, we observed a significantly higher relative abundance of *Erysipelatoclostridiaceae* and *Streptococcaceae* in samples from the Adadle *woreda* compared to any of the other samples included in the analysis (Fig. 3D, *p* value < 0.05). In the primer set 1 dataset, samples coming from the Adadle *woreda* had a higher abundance of *Bifidobacteriaceae* and *Lactobacillaceae* compared to samples coming from CAR and Madagascar (Fig. 3D, *p* value < 0.05). Using SIAMCAT^30^ and LefSe^31^ analysis, we confirmed the association between the higher abundance of the bacterial families and lifestyle (Supplementary Fig. S6).

Altogether the 16S rRNA gene amplicon sequencing data indicate that children living in the Adadle *woreda* have a distinct fecal microbiota composition compared with children of the same age living in different regions of the world. Children from the Adadle region are closer to children coming from transitional communities with lower alpha diversity and lower relative abundance of *Prevotellaceae* than children adopting a similar traditional lifestyle or children from industrialized countries.

### Shotgun metagenomic sequencing confirms distinct fecal microbiota composition

To confirm the amplicon sequencing taxonomic composition trends and account for any primer bias, we used mOTUs2^32^ and MetaPhlan3^33^ taxonomic profilers on the 15 samples sent for shotgun metagenomic sequencing. A total of 2,698,693,772 reads passed fastp^34^ filtering, with an average of 179,912,918 ± 72,371,443 reads per samples. Using mOTUs2, for the Adadle *woreda* dataset, 787 metagenomic-based operational taxonomic units (mOTUs) were assigned to the kingdom of *Bacteria* and accounted for 95.6% of the mapped reads while 4.3% of the reads were unmapped to any species and less than 1% of the reads were assigned to unknown cellular organisms. The 787 mOTUs belonged to 387 known and 56 unknown bacterial species divided in 14 phyla, 82 families and 165 genera (Supplementary data 3). Using MetaPhlAn3 for profiling the microbial community, 349 species were assigned to *Bacteria*, divided in 8 phyla, 62 families and 129 genera (Supplementary data 3). We observed no major differences between MetaPhlAn3 and mOTUs2 profiles at different taxonomic levels (Supplementary Fig. S7A, B). Further, we observed the same trends in the taxonomic composition at the family level of the 6 samples sequenced using both primer sets and by shotgun metagenomic sequencing (Supplementary Fig. S8).

Moreover, using the number of assigned reads in mOTUs2 profiler, we compared the bacterial composition of the samples from the Adadle *woreda* with samples from other communities adopting differing lifestyles (Table 2, Supplementary Fig. S9A, Supplementary data 3). Notably, using PCoA of Bray–Curtis’s distance, we confirmed the previous results from amplicon sequencing that samples from the Adadle *woreda* clustered away from all the other samples on the first and second components (Supplementary Fig. S9C, Supplementary data 7). In addition, Ward’s linkage method for hierarchical clustering at species level resulted in the same two clusters (*P*+ and *P−*) (Supplementary Fig. S9D). Samples from the Adadle *woreda* clustered uniquely in the low *Prevotella* abundance cluster (*P−*), similarly to samples from Lima, Peru (100% in *P−*) and the USA (89.7% in *P−*). Moreover, the clusters repartition was significantly different (χ^2^ test *p* value < *0.05*, Supplementary data 7) from samples from Tanzania (54.5% in *P*+), traditional Peruvian communities (60% in *P*+), Zimbabwe (78.9% in *P*+) and El Salvador (80% in *P*+). Finally, we observed lower relative abundance of both BloSSUM and VANISH taxa compared to children from industrialized countries and traditional communities, respectively (Supplementary Fig. S9B, *p* value  < 0.05) and high relative abundance of *Lactobacillaceae, Bifidobacteriaceae, Erysipelotrichaceae* and *Streptococcaceae* (Supplementary Fig. S9B).

Overall, these results show that the observed fecal microbiota composition was robust across all sequencing methods and taxonomic assignment tools and confirmed the distinctive bacterial composition of the fecal samples of children from the Adadle *woreda* in Ethiopia.

### Samples from the Adadle *woreda* enriched in pathways reflecting dietary habits

To explore the functional profile of the children’s fecal microbiota, we used HUMAnN3 to predict the abundance of microbial metabolic pathways present in our shotgun metagenomic dataset^33^. HUMAnN3 detected 1,400,457 evolutionary-related protein-coding sequences grouped in gene families which mapped to 490 known microbial pathways (Supplementary data 3). The total abundance of genes that contributed to a pathway accounted on average for 5.97% while the ones that did not contribute to any known pathways accounted for 69.57%. Additionally, the total abundance of reads unmapped to any known gene accounted on average for 24.46% (Supplementary Fig. S10). Out of the 490 detected pathways, 26 were uniquely found in samples from the Adadle *woreda*. In samples from other communities, we found 23 additional pathways not observed in samples from the Adadle *woreda*. Finally, 268 out of 490 pathways were detected in every sample from the Adadle *woreda*, among these pathways 95 were detected in all samples from both agropastoralists from the Adadle *woreda* and all other populations. Metabolic pathways were grouped in 7 superclass categories 1, with biosynthesis being the most abundant superclass (4.35 ± 0.0044%), followed by degradation/utilization/assimilation (0.96 ± 0.0021%) and generation of precursor metabolites and energy (0.55 ± 0.00062%) (Supplementary Fig. S10, Supplementary data 7). Further, pathways were classified in 46 superclass categories 2, with the first 5 most abundant being amino acid biosynthesis (1.20 ± 0.0016%), nucleoside and nucleotide biosynthesis (0.84 ± 0.0014%), cofactor, carrier, and vitamin biosynthesis (0.77 ± 0.00079%), carbohydrate biosynthesis (0.43 ± 0.00052%), followed by carbohydrate degradation (0.38 ± 0.00091%) (Supplementary Fig. S10, Supplementary data 7).

**Figure 4:**
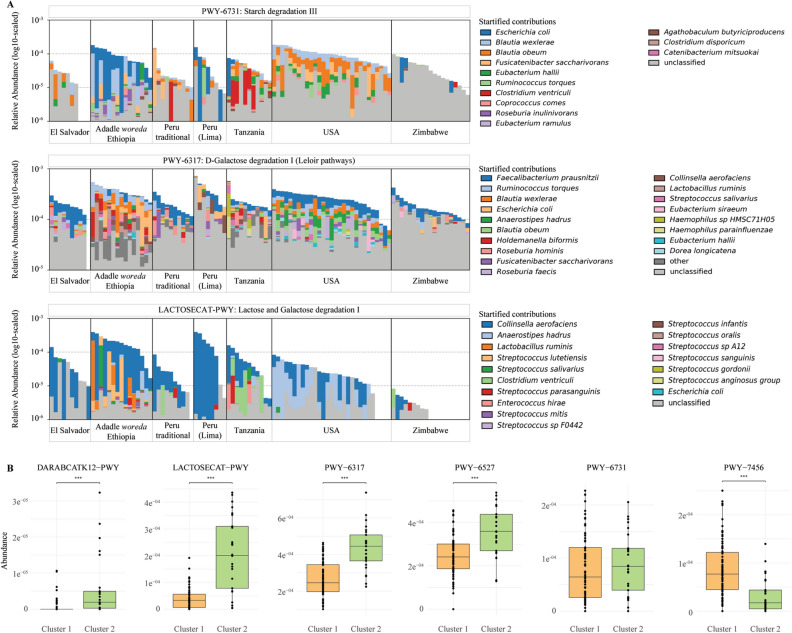
Enrichment of carbohydrate degradation pathways in the different clusters. (**A**) Stacked bar plots of the log10 of the relative abundance of species contributing to pathways PWY-6731, PWY-6317 and LACTOSECAT-PWY. (**B**) Carbohydrate degradation pathways enriched in the different clusters. DARABCATK12-PWY: D-arabinose degradation I, LACTOSECAT-PWY: lactose and galactose degradation I, PWY-6317: galactose degradation I, PWY-6527: stachyose degradation, PWY-6731: starch degradation III, PWY-7456: mannan degradation. Hierarchical clustering of the samples using Ward’s linkage method. Pathways enrichment in the clusters tested with MaAsLin2 at q-value < 0.05. (***q < 0.05). N = 102.

In addition, we noticed different species contributing to metabolic pathways in the feces of geographically distant communities. Amongst the 95 detected species, 20 contributed to a metabolic pathway in all communities, such as *Escherichia coli,* or *Ruminoccoccus torques*. While 39 were involved uniquely in samples from the Adadle *woreda*, including *Bifidobacterium catenulatum, Bifidobacterium longum* and *Lactobacillus ruminis,* 25 species, notably *Blautia obeum* and *Treponema succinifaciens,* were not involved in any of the metabolic pathways found in the samples from the Adadle *woreda* (Supplementary Fig. S11). Additionally, we noted a high diversity of *Streptococcus* species contributing to metabolic pathways in samples from the Adadle *woreda*. These species were notably involved in carbohydrate degradation pathways, including starch (PWY-6731), lactose (LACTOSECAT-PWY) and galactose (PWY-6317), (Fig. 4) as well as stachyose (PWY-6527), sucrose (PWY-5384, PWY-621), and glycogen (GLYCOCAT-PWY, PWY-5941) (Supplementary Fig. S12, Supplementary data 3).

Last, we used Ward’s linkage method for hierarchical clustering of the samples and identified two clusters (*Clust1* and *Clust2*) (Supplementary Fig. S13). Samples from the Adadle *woreda* (14/15) and from the transitional community of Lima, Peru (8/8) clustered mostly in *Clust2* and samples from the USA (29/29), El Salvador (9/10), and the traditional populations of Tanzania (10/11), Zimbabwe (18/19) and Peru (10/10) clustered mostly in *Clust1* (Supplementary data 7). Out of the 513 pathways, we identified 228 that showed significant differences in abundance between the two clusters (Wilcoxon rank test at a significance threshold of 0.05 with Bonferroni correction for multiple comparisons, Supplementary data 7). Of these, 6 were enriched in *Clust*1 and 222 were enriched in *Clust2*.

Amino acid biosynthesis superclass 2 was significantly different between the two clusters (*p* value = 1.272e^−10^) but contrasting results were observed at the pathway level with 37 out of 47 pathways related to amino acid biosynthesis enriched in *Clust2* (Supplementary Fig. S14). Out of the 27 carbohydrate degradation pathways, only mannan degradation (PWY-7456, *p* value = 7.367e^−4^) was enriched in *Clust1* while 12 pathways were enriched in *Clust2*, including the degradation of lactose (LACTOSECAT-PWY, *p* value  = 3.696e^−5^), galactose (PWY-6317, *p* value = 2.221e^−5^), d-arabinose (DARABCATK12-PWY, *p* value = 4.17e^−8^), and stachyose (PWY-6527, *p* value = 2.143e^−5^). Starch degradation was not enriched in either of the clusters (PWY-6731, *p* value > 0.05) (Fig. 4, Supplementary data 7).

### Resistome

**Figure 5:**
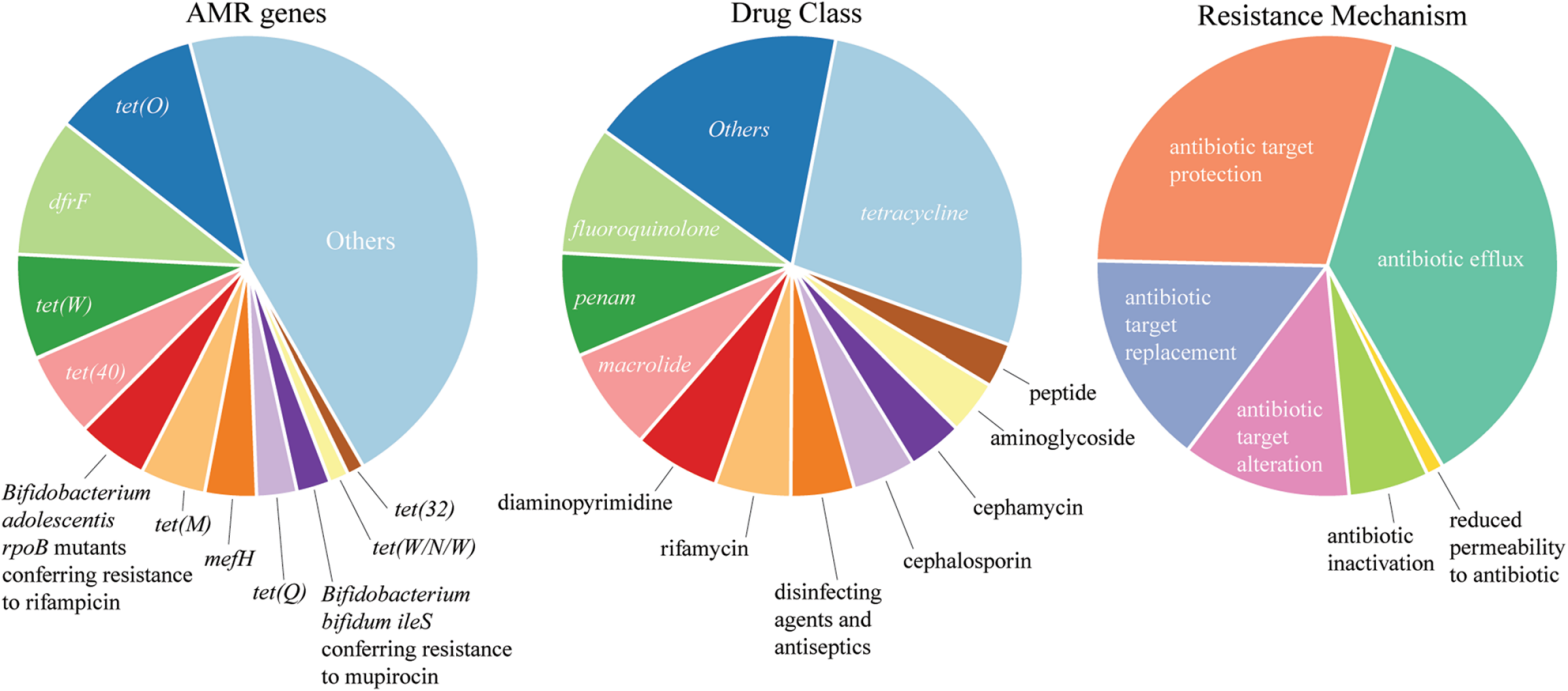
Overview of the resistome in the feces of children living in the Adadle *woreda*. From left to right: Antimicrobial Resistance genes (AMR). Drug classes to which AMR genes confers resistance. Resistance mechanism given by the AMR genes. *tet* tetracycline, *dfr* dihydrofolate reductase, *rpoB* rifamycin-resistant beta-subunit of RNA polymerase, *mef* major facilitator superfamily antibiotic efflux pump, *ileS* isoleucyl-tRNA synthetase, *penam* penicillin. N = 15.

Finally, to assess for the presence of putative resistance genes in the gut microbiome of children from the Adadle *woreda*, we quantified the antibiotic resistome by mapping genes to the reference database CARD^35^. Among the 15 samples, we found 166 putative antimicrobial resistance (AMR) genes, potentially conferring resistance to 29 functional drug classes (Supplementary data 3). Antibiotic efflux was the most frequently detected encoded resistance mechanism, followed by antibiotic target protection, antibiotic target replacement, antibiotic target alteration, antibiotic inactivation, and reduced permeability (Fig. 5). We observed that AMR genes predicted to confer resistance against tetracycline were the most common, followed by AMR genes related to resistance against fluoroquinolones, penams (penicillin), and macrolides (Fig. 5). The most abundant genes were *tet(O)*, followed by *dfrF*, *tet(W)*, *tet(40), and Bifidobacterium adolescentis rpoB* mutants conferring resistance to rifampicin (Fig. 5).

In samples from other communities, genes, such as *tet(O)*, *tet(Q),* and *tet(W),* conferring resistance against tetracycline were consistently the most commonly detected resistance genes. Resistance against macrolide and streptogramin antibiotics and specific AMR genes, such as *cfxA6* and *cfxA2,* related to resistance to cephamycin were also frequent in samples from all communities. Additionally, in samples from the transitional community of Lima, resistance against rifamycin and mupirocin-like antibiotics conferred by *Bifidobacterium adolescentis rpoB* and *Bifidobacterium bifidum ileS*, respectively, were the most common resistance genes detected (Supplementary data 7). While samples from the Adadle *woreda* seem to cluster away from the other communities on the x-axis of the PCoA of the Jaccard distance, our data suggests that the position of the samples is correlated with the sequencing depth (Supplementary Fig. S15). Additionally, the presence and absence of the putative AMR genes and the drug classes were tested using generalized linear models, but none were significantly different between the communities.

## Discussion

Here, we characterized the fecal microbiota composition and function of 59 agropastoral children, aged 2–5 years, from the Adadle *woreda* of the Somali Regional State of Ethiopia. With the use of 16S rRNA gene amplicon and shotgun metagenomic sequencing, our data suggest that these children harbor a specific microbiome. This community composition may reflect their dietary habits and that their microbiota is closer to that of children from transitional communities than to that of children living similar traditional lifestyles.

The observed difference in the microbiota composition between the agropastoralist children from the Adadle *woreda* and children from other communities is in line with the diet adopted by the population of the Adadle region. Their diet has an extremely low variety and consists mainly of milk and to a lower extent of starch-rich foods such as rice and wheat. None of the children had meat or fish in the last 24 h prior to sampling. Only a few children consumed tomatoes or onions but no other vegetables or fruits were reported as being consumed the day prior sample collection^27^. This is reflected in the composition of the fecal microbiota with, notably, a higher abundance of *Streptococcaceae*, *Bifidobacteriaceae*, *Lactobacillaceae* as well as *Akkermansiaceae* and a lower abundance of *Bacteroidaceae, Prevotellaceae, Succinivibrionaceae*, and *Spirochaetes*.

The higher relative abundance *Akkermansiaceae*, *Bifidobacteriaceae* and *Lactobacillaceae* is most likely due to the high consumption of milk in our study group. Indeed, *Akkermansiaceae* was recently shown to thrive on milk oligosaccharides in vitro^36^ and *Bifidobacteriaceae* and *Lactobacillaceae* are well known to be boosted by the consumption of dairy products^11^. *Streptococci* thrive on simple sugars^37,38^ and their high abundance might therefore be associated with the consumption of wheat and rice, one of the main food items consumed by the agropastoralists besides milk^27^. Additionally, metagenomic analysis of the bacterial community of Ethiopian traditional fermented camel milk^39^, a commonly consumed milk in the Adadle region alongside milk from other livestock^40^, revealed that species of *Streptococci* were amongst the most abundant and most prevalent detected bacteria. This may further explain their high abundance in fecal samples from the Adadle *woreda*. Interestingly, two *Bifidobacterium* species, *Lactobacillus ruminis,* and diverse *Streptococcus* species were found to be contributing to the degradation of carbohydrates. This suggests a primordial role of *Streptococci* in overall community metabolism in the samples from the Adadle *woreda*. Pathways for the degradation of lactose, one of the main constituent of milk^41^, and d-galactose, one of the mono-saccharides forming lactose and stachyose, as well as simple carbohydrates such as d-arabinose and fucose were enriched in fecal samples from the Adadle *woreda*. These pathways likely reflect the abundant consumption of milk and food products composed of simple sugars in this community.

Species of the *Bacteroidaceae* family have been previously associated with a higher consumption of animal fat and protein in westernized societies^18,19,42^. The very low levels in *Bacteroidaceae* observed in our study group are likely linked to the low consumption of these food items. The enrichment of metabolic pathways related to amino acid biosynthesis observed in our study might be linked to the low protein consumption by the agropastoral children from the Adadle *woreda*. However, little is known on this subject and more investigations would be needed. In contrast with earlier studies^15,42–44^, we did not observe an increased abundance of *Prevotellaceae, Succinivibrionaceae*, or *Spirochaetes,* which were previously associated with a traditional lifestyle^17^. This findings suggest that these taxa are likely dependent on the fiber-rich vegetables and fruit-based diets often observed in other traditional communities^18^. In our study, we observed an extremely low abundance of *Prevotella*, which contrasts with other studies of the fecal microbiome of populations with a traditional lifestyle^14–16,19,20,45^. Interestingly, *Treponema succinifaciens*, a member of the *Spirochaetes* family, was not found to be involved in any metabolic pathway in samples from the Adadle *woreda* but found to be involved in the degradation of D-galactose in samples from El Salvador and traditional communities of Peru, Tanzania, and Zimbabwe. This virtual absence of *Prevotella*, *Succinivibrionaceae* and *Spirochaetes* is probably a result from the adaptation of the microbiota to a diet poor in fiber and complex carbohydrates in the agropastoral children from the Adadle *woreda*.

The high abundance of *Erysipelotrichaceae* and *Erysipelatoclostridiaceae* is of surprise, as these families have been shown to increase upon consumption of a high-fat, westernized diet in mice^46^. Additionally, members of these families have been found in the gut microbiota of cattle, notably in Mongolia^47^. We hypothesize that the higher level of these taxa might be due to the closeness of the children with cattle. Further, the consumption of camel milk, in which fat matter is one of the main component,^41^ could promote the growth of these taxa.

Additionally, we assessed the presence of putative antibiotic resistances genes in the feces of children living in the Adadle region. AMR genes mapped to the CARD database and predicted to confer resistance to antibiotic such as tetracycline, fluoroquinolones, penams (penicillin) and macrolides were notably detected, and we noticed variations in the pool of putative AMR genes in the different communities, but these differences were not statistically significant. The observed AMR genes were predicted by mapping against the CARD database, representing known genes. Other complementary machine learning methods as well as structural approaches should be used in future studies to predict putative AMR genes. Additionally, the expression of the observed AMR genes would need to be evaluated experimentally to confirm the resistance potential found in the feces of children from the Adadle *woreda*.

Even though the children from the Adadle *woreda* follow a traditional agropastoral lifestyle, we observed that their fecal microbiota composition and function was significantly different than the one in children from other traditional communities. In recent years, numerous studies have highlighted the associations between bacterial taxa and specific lifestyles^14–16,19,20,45,48–50^. In accordance with these studies, we showed that the agropastoral way of living of the children from the Adadle region shapes their microbiome. However, we observed different bacterial taxa being more prevalent and abundant than the usual taxa found to be associated with traditional communities. These differences are likely due to the high specificity and limited variety in the diet consumed by the children of the Adadle *woreda*. These findings highlight the importance of including dietary information in studies aimed to characterize the intestinal microbiota. Further, additional factors such as the presence of parasitic infections or periods of dietary restrictions might also influence the microbiota composition in the Adadle region^27,28^.

Our study has several notable strengths: to the best of our knowledge, it is the first study to describe the fecal microbiota in the Somali Regional State of Ethiopia. Further, the specific diet, dominated by milk products and starch-rich foods, is widely different from the diet of children previously studied. Last, using both 16S rRNA gene amplicon sequencing using two different primer pairs as well as shotgun metagenomic sequencing allow us to robustly profile the microbiota composition of the children from the Adadle *woreda*. However, as expected, using different sequencing methods and profiling tools revealed slightly differing results depending on the chosen method and tools. While the comparison between groups were not influenced by the profiling tools and sequencing methods, the description of the microbiota composition varied in abundance. As any study including secondary data analysis, our study has a few limitations. These include bias introduced by the fact that we were not able to control for sampling, storage, and DNA extraction methods in the data retrieved from public repositories. Further, the small sample size in our study group could influence the observation made on the microbiota composition and function of children from the Adadle *woreda*. This point should be addressed in future studies with larger sample sizes.

In conclusion, this study reveals a unique fecal microbiota composition and function of agropastoral children living in the Adadle *woreda* in the Somali regional state of Ethiopia. This unique microbial profile is likely influenced by their specific and low-diversity diet. Our study highlights the need to further understand the microbial composition of communities with different lifestyles and geographic origins in a bid to improve our knowledge on microbiota dynamics and the associated health outcomes. Such advances could ultimately be used to develop personalized and effective treatments for dysbiosis-associated diseases. This study sets a baseline for further research assessing dysbiotic microbiota which may occur during regular periods of malnutrition in the Somali regional state. Future research may also characterize livestock microbiota, as agropastoral communities live in very close proximity to their livestock and under poor sanitation and hygiene conditions. A One Health approach characterizing the microbiome in an interconnected manner will be crucial to better understand the specific profile found in this population.

## Methods

### Cohort/study population

This study included feces from 59 children aged 2–5 years, sampled in the context of a previous study on parasitic infection and micronutrient status conducted in the Adadle *woreda* (district) of the Somali regional state of Ethiopia, in the dry season between July and September 2016^27^. This region is mostly inhabited by pastoral and agropastoral Muslims. The original cross-sectional cohort study included 387 subjects from pastoral and agropastoral households, but only a small fraction corresponded to the age group selected for (2–5 years), had a height for age z-score score above − 1.5, and had a fecal sample available for DNA extraction and microbiota analysis (Supplementary Fig. S16). In total, 59 samples from children living in 3 different kebele (municipalities), Gabal, Higlo and Buursaredo were sent for 16S rRNA amplicon sequencing and 15 for whole-genome shotgun metagenomic sequencing (Supplementary Fig. S15).

### Sample collection, DNA extraction and sequencing

Stool samples were collected as described by Osman et al.^27^. Briefly, plastic containers with detailed instructions for collection of fresh stool sample were given to mothers or caregivers and collected the same day of sample preparation and freezing at the local health facility. DNA was extracted using a commercial kit (QiaAmp DNA Mini Kit, Qiagen) with an additional bead-beating step according to a pre-established protocol^51^. In brief, 100 mg of feces were homogenized by bead-beating with 0.7–1.2 mm Granat beads (BioLabProducts GmbH) in 250 μl 2% Polyvinylpolypyrrolidone (PVPP) buffer (Sigma Aldrich). Then DNA extraction steps were conducted as indicated by the DNA extraction kit’s manufacturer.

Extracted DNA samples were shipped to two different sequencing service providers (Microbiome Insights, Vancouver, Canada and Integrated Resource, Dalhousie University, Halifax, Canada) where library generation and sequencing were performed. At Microbiome Insights, library preparation was performed using the primer set v4.SA501-v4.SA508 (forward) and v4.SA701-v4.SA712 (reverse) (referred as primer set 1), targeting the 16S V4 region^52^. The amplicon library was sequenced on a MiSeq using the MISeq 500 Cycle V2 Reagent Kit (2 × 250 bp paired-end). At Dalhousie University, library preparation was performed using the 515F/806R primer pair (referred as primer set 2) which amplifies the V4 region of the 16S rRNA gene^53,54^. The amplicon library was sequenced on Illumina MiSeq (2 × 300 bp paired-end) using V3 chemistry.

Whole-genome shotgun metagenomic sequencing was performed by Eurofins Genomics (Eurofins Genomics Europe Sequencing GmbH, Konstanz, Germany) using the Illumina HiSeq (Sequence mode NovaSeq 6000 S2 PE150 XP).

### Secondary data analysis of previously published studies

Additional sequences for reference groups were sourced from either the Afribiota project^55^ (Table 2, Supplementary data 2, using primer set 1 with primers v4.SA501-v4.SA508/v4.SA701-v4.SA712) as well as several additional published studies (Table 2, Supplementary data 2, using primer set 2 with primers 515F/806R). The final 16S rRNA amplicon sequencing dataset included in addition to the 59 sequences from the Adadle *woreda*, 705 fecal samples from the Afribiota project (Primer set 1) and 679 fecal samples from other previously published studies (Primer set 2) described in Table 2. The shotgun metagenomic dataset included in addition to the 15 samples from the Adadle *woreda*, 87 samples from previously published studies (Table 2, Supplementary data 2).

Lifestyle classifications of the different populations are based on the original publications. Briefly, hunter-gatherers, pastoralists, agropastoralists and agriculturalists were classified as traditional; populations living in rural, peri-urban and urban area of low- and middle-income countries as transitional; populations from urban North American and European cities as industrial. Samples from the Adadle *woreda* are classified in this manuscript as Adadle agropastoralism in order to separate our samples from samples from other traditional communities.

**Table 2:** Additional sequences sourced from previously published studies.

Country	Population/region	Age range (years)	Lifestyle	N	Study
16S rRNA gene amplicon sequencing samples					
Primer set 1				705	
Central African Republic	Bangui	2–5	Transitional	274	^55^
Madagascar	Antananarivo	2–5	Transitional	431	^55^
Primer set 2				679	
Bangladesh	Dhaka (Mirpur)	3–4	Transitional	51	^56^
Cameroon	Baka, Bantu	4–5	Traditional	5	^57^
China	Nagqu, Hongyuan, Gangcha, Lhasa, Tianzhu, Gannan (Tibet)	2–5	Traditional	24	^58^
El Salvador	South of San Salvador	2–5	Transitional	29	^59^
Malawi	Mayaka, Mbiza	2–3	Traditional	13	^16^
Peru	Matses, Tunapuco	2–5	Traditional	11	^15^
	Lima	2–5	Transitional	8	^59^
Sweden	Halmstad	3–4	Industrial	335	^26^
Tanzania	Hadza (Sengeli, Hukamako)	3–5	Traditional	35	^42^
USA	St Louis, Philadelphia, Boulder	2–5	Industrial	8	^16^
	Los Angeles	2–5	Industrial	141	^60^
Venezuela	Platanillal, Coromoto	2–5	Traditional	19	^16^
Shotgun metagenomic sequencing samples				87	
Peru	Matses, Tunapuco	2–5	Traditional	10	^15^
	Lima	2–5	Transitional	8	^59^
Tanzania	Hadza	2–4	Traditional	11	^23^
El Salvador	South of San Salvador	2–5	Transitional	10	^59^
Zimbabwe	Chihuri, Mupfure	2–5	Traditional	19	^61^
USA	Rhode Island	2–5	Industrial	29	^62^

### Bioinformatic analysis of the 16S rRNA gene amplicon sequences

Bioinformatic analysis was performed using DADA2 (v1.22) according to a previously well-described standard pipeline^63^. Briefly, retrieved sequences were filtered and trimmed based on the sequencing quality (240nt forward reads, 220nt reverse reads) and paired-end reads were merged after dereplication and sample inference (Supplementary data 2). Taxonomy was assigned by matching the sequences to the Silva reference database (v138.1)^64^. Sequences alignment and phylogenetic tree construction were performed using DECIPHER (v2.22.0)^65^ and phangorn (v2.10.0)^66^ packages. Samples with less than 5′000 reads were excluded from the analysis. Further, mitochondrial DNA, chloroplasts as well as sequences with an assignment not belonging to the kingdom of *Bacteria* and *Archaea* were removed. Out of the 59 samples sent for amplicon sequencing, 54 using primer set 1 and 13 using primer set 2 passed quality filtering and inclusion criteria for analysis. Each primer dataset was processed separately, and the final taxonomy and sequence count tables were then joined for the final analysis (Supplementary data 3, 4, 5).

Raw sequences sourced from other studies were processed as described above in DADA2^63^. Individual studies were processed independently until merging of the sequence tables for joint chimeras’ removal and taxonomy assignment. The sequences that passed the quality control are summarized in Supplementary data 2. Resulting amplicon sequence variants (ASVs) tables and taxonomy tables were filtered and processed as described above.

To correct for differences in sequencing depth for alpha and beta diversity analysis, samples were rarefied to 5′000 reads (Supplementary Fig. S17). Alpha diversity (species diversity) was measured using the Faith’s phylogenetic diversity. For β-diversity analysis, singletons were removed, and logarithmic transformation was applied for principal coordinates analysis (PCoA) of WeightedUniFrac, Bray–Curtis, Jaccard and Generalized UniFrac distances at species level. Hierarchical clustering was performed using Euclidian distance and Ward’s linkage method. The Calinski–Harabasz’s index was calculated to obtain the optimal number of clusters to split the dendrogram resulting from the hierarchical clustering. Differential abundance analysis was performed using SIAMCAT (v1.14.0)^30^ and LefSe^31^ from the microbiomeMarker package (v1.0.2)^67^.

### Bioinformatic analysis of whole-genome shotgun metagenomic sequences

Shotgun metagenomic data were first treated with fastp (v0.20.1)^34^ for quality control, trimming of adapters and quality filtering. Taxonomic assignation was performed using mOTUs profiler version 2^32^ with the output in number of reads (Supplementary data 3) to corroborate the 16S rRNA taxonomic profile. Mitochondrial DNA, chloroplasts, as well as sequences with an assignment not belonging to the kingdom of *Bacteria* and *Archaea* were removed from the abundance table.

Profiling of microbial metabolic pathways was performed with HUMAnN 3.0 (v3.0.1)^33^ using the taxonomy abundance table obtained with MetaPhlAn3 taxonomic profiler^33^, the full ChocoPhlAn database (v296_201901b) from the BioBakery3^33^ and the UniProt database (UniRef90_annotated_v201901b_full)^68^. The utility script humann_renorm_table with output in relative abundance was used to normalize the default HUMAnN’s output reads per kilobase (RPK) and correct for different samples sequencing depths (Supplementary data 3). Metabolic pathways were classified using the MetaCyc Metabolic Pathway Database (MetaCyc 19.1) at the superclass 1, superclass 2 and pathways levels^69^.

To identify antimicrobial resistance genes, the Resistance Gene Identifier (RGI *bwt* v6.0.0) was used to map reads on the Comprehensive Antibiotic Resistance Database’s protein homolog model (CARD, v3.2.5, Supplementary data 3)^35^. Results were filtered for genes with at least 100 mapped reads and 80% coverage. Further, reads per kilobase million (RPKM) was used to correct for gene length and sequencing depth efforts.

### Biostatistics analysis

Biostatistical analysis was performed in the R environment and language (v4.1.2, R Core Team, 2021) using the packages phyloseq (v1.38.0)^70^, vegan (v2.5-7)^71^, microbiomeMarker (v1.0.2)^67^, microbiome (v1.6.5)^72^, ape (v5.6-1)^73^, picante (v1.8.2)^74^ and clusterCrit (v1.2.8)^75^. Data visualizations were realized with packages RColorBrewer (v1.1-3)^76^, ComplexHeatmap (v2.13.1)^77^ and ggplot2 (v3.3.5)^78^. The detailed R-scripts can be found on github (https://github.com/VonaeschLabUNIL/Pastobiome).

Relative abundance differences and Faith’s phylogenetic diversity differences were tested using Wilcoxon rank test at a significance threshold of 0.05 with Bonferroni correction for multiple comparisons. Differential abundance was analyzed using SIAMCAT (v1.14.0)^30^ and LefSe^31^ from the microbiomeMarker package (v1.0.2)^67^. A pseudo-count of 1e^-4^% was added to relative abundances of 0 for logarithmic transformation. Analysis of variance using distance matrix was performed using ADONIS2 from vegan (v2.5-7)^71^ package with Benjamini–Hochberg correction for multiple comparisons. Enrichment of pathways was analyzed using MaAsLin2 (v1.8.0)^79^ and SIAMCAT (v1.14.0)^30^.

### Ethical approval

The study was conducted according to the declaration of Helsinki, and ethical clearance was obtained from the Review Committee of the University of Jigjiga in Ethiopia (JJU-RERC 002/2016) and the Swiss Ethics Committee of Northwest and Central Switzerland (Ethikkommision Nordwest- und Zentralschweiz; EKNZ BASEC UBEreq. 2016-00204). A material transfer agreement was established by the Food, Medicine and Health Care Authority of Ethiopia for the shipment of fecal samples from Ethiopia to Switzerland. All the parents/caregivers of the participating children gave oral and written consent prior to the study enrollment of their children.

### Supplementary Information

Supplementary Information 1.

Supplementary Information 2.

Supplementary Information 3.

Supplementary Information 4.

Supplementary Information 5.

Supplementary Information 6.

Supplementary Information 7.

## Data Availability

All raw data included in this study have been uploaded to NCBI Sequence Read Archives under accession number PRJEB61656. The datasets analyzed during the current study are available in the NCBI Sequence Read Archives repository under accession numbers: PRJEB48119, PRJNA547591, PRJNA392012, PRJNA381333, PRJEB13051, PRJEB3079, PRJEB38986, PRJNA300541, PRJEB27068, PRJEB27517, PRJNA521455, or on figshare repository, https://doi.org/10.6084/m9.figshare.7011272.v3.

## Abbreviations

CAR: Central African Republic
VANISH: Volatile and/or associated negatively with industrialized societies of humans
BloSSUM: Bloom or selected in societies of urbanization/modernization
Primer set 1: 16S rRNA gene primer v4.SA501-v4.SA508 and v4.SA701-v4.SA712 targeting the V4 region
Primer set 2: 16S rRNA gene primer 515F and 806R targeting the V4 region
ASVs: Amplicon sequence variants
PD: Faith’s phylogenetic diversity
PCoA: Principal coordinates analysis
WUF: Weighted UniFrac
mOTUs: Metagenomic-based operational taxonomic units
AMR: Antimicrobial resistance
