# Supplementary material for: Gut microbiomes of agropastoral children from the Adadle region of Ethiopia reflect their unique dietary habits
Source: Sci Rep. 2023 Dec 1;13:21342. doi: 10.1038/s41598-023-47748-8 (PMC10696028; doi:10.1038/s41598-023-47748-8)
Supplement: Supplementary file 1 — Supplementary Information 1. [file 41598_2023_47748_MOESM1_ESM.pdf]

Gut microbiomes of agropastoral children from the Adadle region of Ethiopia show a unique bacterial composition reflecting their dietary habits

Simon Yersin, Julian R. Garneau, Pierre H. H. Schneeberger, Kadra A. Osman, Colin I. Cercamondi, Abdifatah M. Muhummed, Rea Tschopp, Jakob Zinsstag, and Pascale Vonaesch

Additional files

Additional file 1: (.pdf) **Supplementary figures:** Supplementary figure S1 to S16.

Additional file 2: (.xls) **Complementary information and metadata**: Complementary information, DADA2 parameters, reads retention and metadata table.

Additional file 3: (.xls) **Taxonomy tables**: Taxonomy tables for primer set 1, primer set 2, mOTUs and MetaPhlan3. HUMAnN3 pathways and RGI tables.

Additional file 4: (.xls) **Primer set 1 ASV table**: Primer set 1 ASV table.

Additional file 5: (.xls) **Primer set 2 ASV table**: Primer set 2 ASV.

Additional file: 6: (.xls) **16S rRNA gene sequencing information and statistical test results:** Sequencing information, phylum and family prevalence and relative abundance and statistical test results. Statistical test results of the comparison of relative abundance and prevalence between the two primer sets.

Additional file 7: (.xls) **Statistical test results for shotgun metagenomic analysis:** Statistical test results for the shotgun metagenomic dataset and list of ARO terms, drug classes and resistance mechanisms.

Supplementary figures

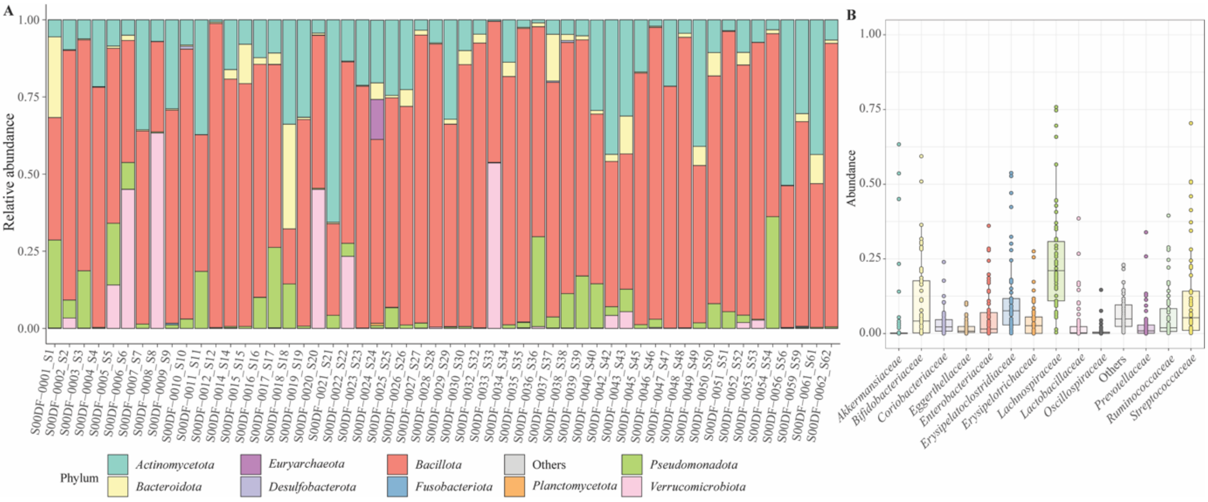

Figure S1: Composition of the fecal microbiota of children living in the Adadle region using a 10% prevalence filter. *(A) Relative abundance of the most abundant phyla for samples from the Adadle woreda. All other phyla are grouped in the Others category. (B) Box plot of the relative abundance of the most abundant bacterial families for samples from Adadle woreda. All other families are cumulated together in the Others category.*

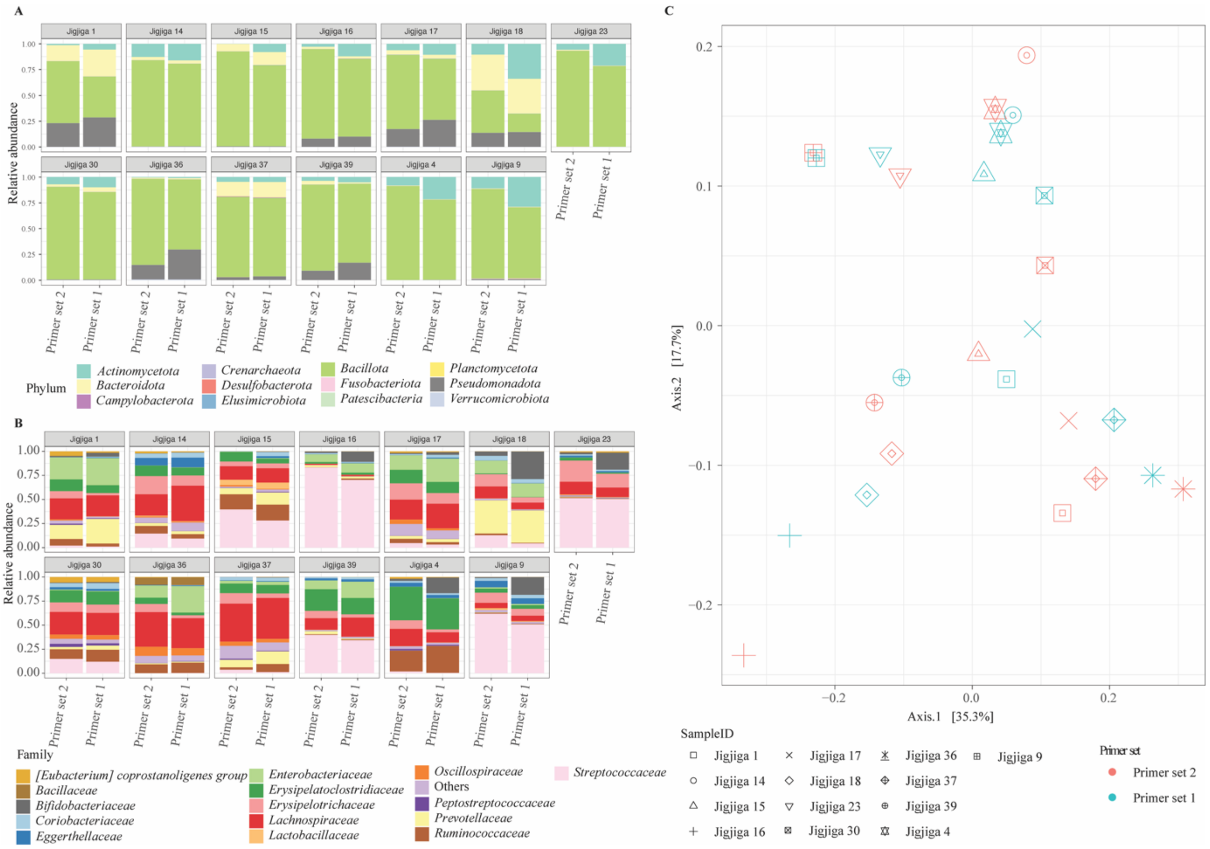

Figure S2: Comparison of the 13 samples sequenced using both datasets. *(A) Stack bar plot of the relative abundance of the phyla comparing samples sequenced with the two primer sets. (B) Stack bar plot at the family level comparing the samples sequenced with the two primer sets. (C) PCoA of Bray-Curtis distance of the samples sequenced with the two primer sets.*

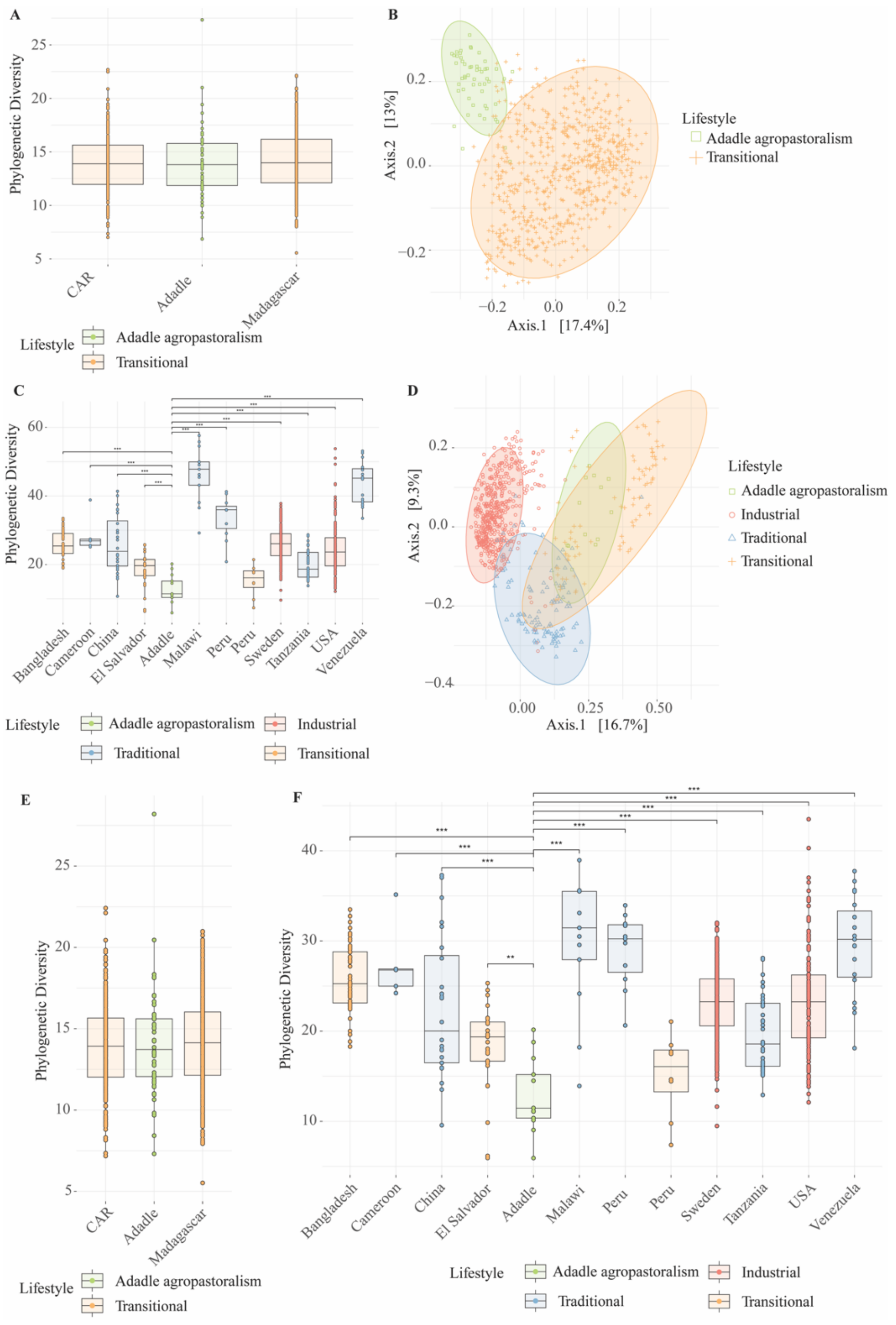

Figure S3: Diversity metrics using a 0.25% abundance filter and multiple rarefaction. *(A) Phylogenetic diversity of the primer set 1 using a 0.25% abundance filter on the dataset. (B) PCoA of the GUniFrac distance of the primer set 1 samples with a 0.25% abundance filter. (C) Phylogenetic diversity of the primer set 2 using a 0.25% abundance filter on the dataset. (D) PCoA of the GUniFrac distance of the primer set 2 samples with a 0.25% abundance filter. (E) Phylogenetic diversity of the primer set 1 using multiple rarefaction (N=100) and calculating the mean phylogenetic diversity of the rarefaction runs. (F) Phylogenetic diversity of the primer set 2 using multiple rarefaction (N=100) and calculating the mean phylogenetic diversity of the rarefaction runs. CAR : Central African Republic.*

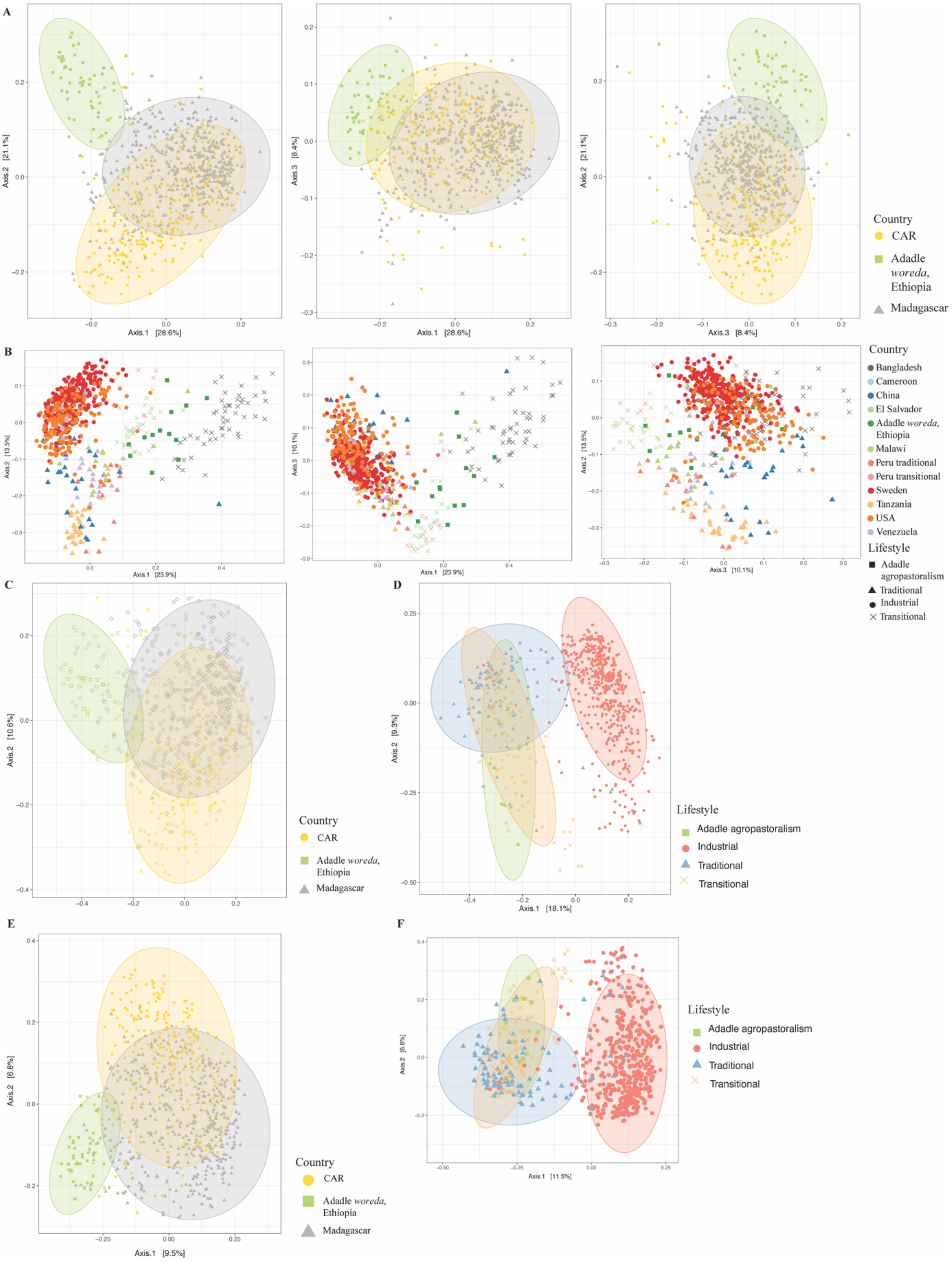

Figure S4: Principal Coordinate Analysis. *(A) Principal Coordinates Analysis (PCoA) of Weighted UniFrac distance on axis 1-2, 1-3 and 1-3 for primer set 1 and (B) primer set 2. (C) PCoA of Bray-Curtis for primer set 1 and (D) primer set 2. (E) PCoA of Jaccard distance for primer set 1 and (F) primer set 2. Primer set 1 are primers v4.SA501-v4.SA508/v4.SA701-v4.SA712. Primer set 2 are primers 515F/806R. CAR : Central African Republic.*

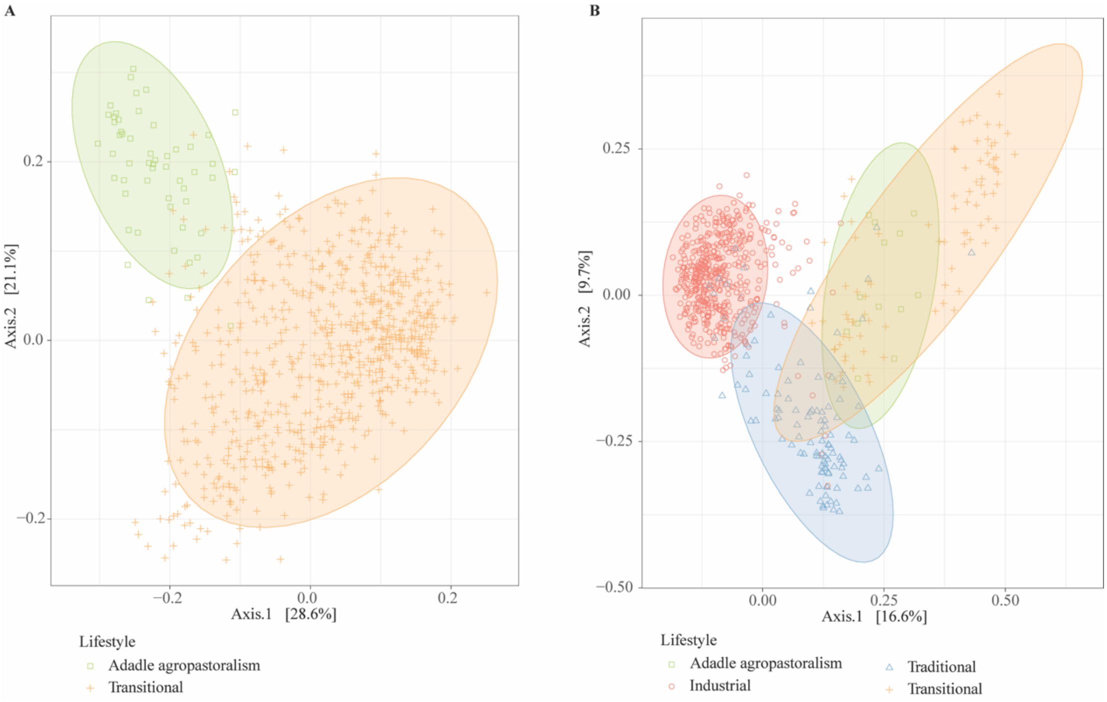

Figure S5: PCoA of the Generalized UniFrac. *(A) PCoA of GUniFrac distance for primer set 1 and (B) primer set 2.*

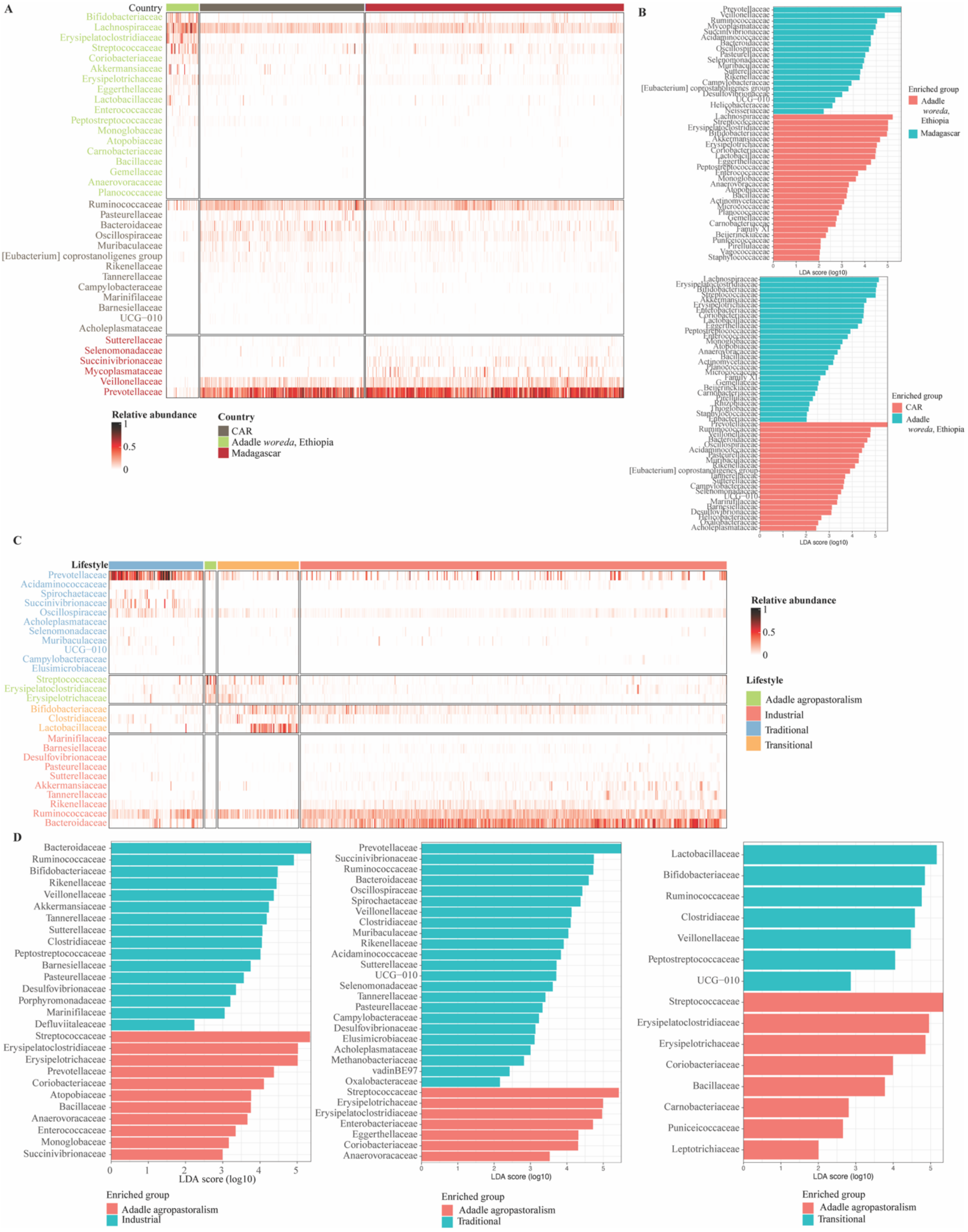

Figure S6: Differential abundance analysis using SIAMCAT and LefSe. *(A) Heatmap of the bacterial families associated with each country, Adadle woreda, Central African Republic (CAR) and Madagascar, using SIAMCAT on primer set 1. (B) LefSe analysis showing bacterial families associated with each country in primer set 1. (C) Heatmap of the bacterial families associated with each lifestyle, using SIAMCAT on primer set 2. (B) LefSe analysis showing bacterial families associated with each lifestyle in primer set 2.*

A

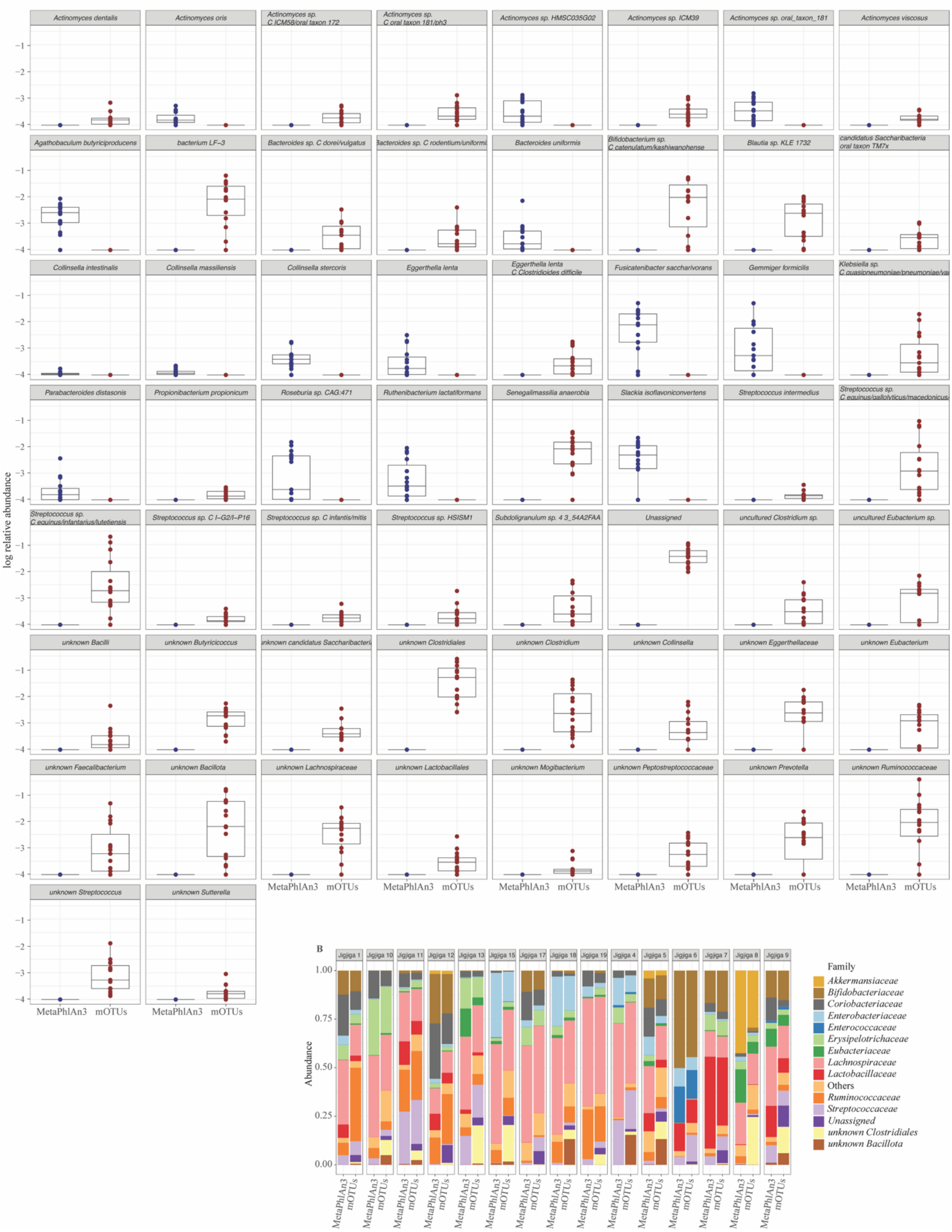

***Figure S7: Comparison of the resulting abundance using MetaPhAn3 and mOTUs2**. (A) Species with a significantly different abundance between the two profiling tools, mOTUs2 and MetaPhlAn3. (B) Stack bar plot at the family level comparing the 15 samples sequenced by shotgun metagenomic and profiled using the two profiling tools.*

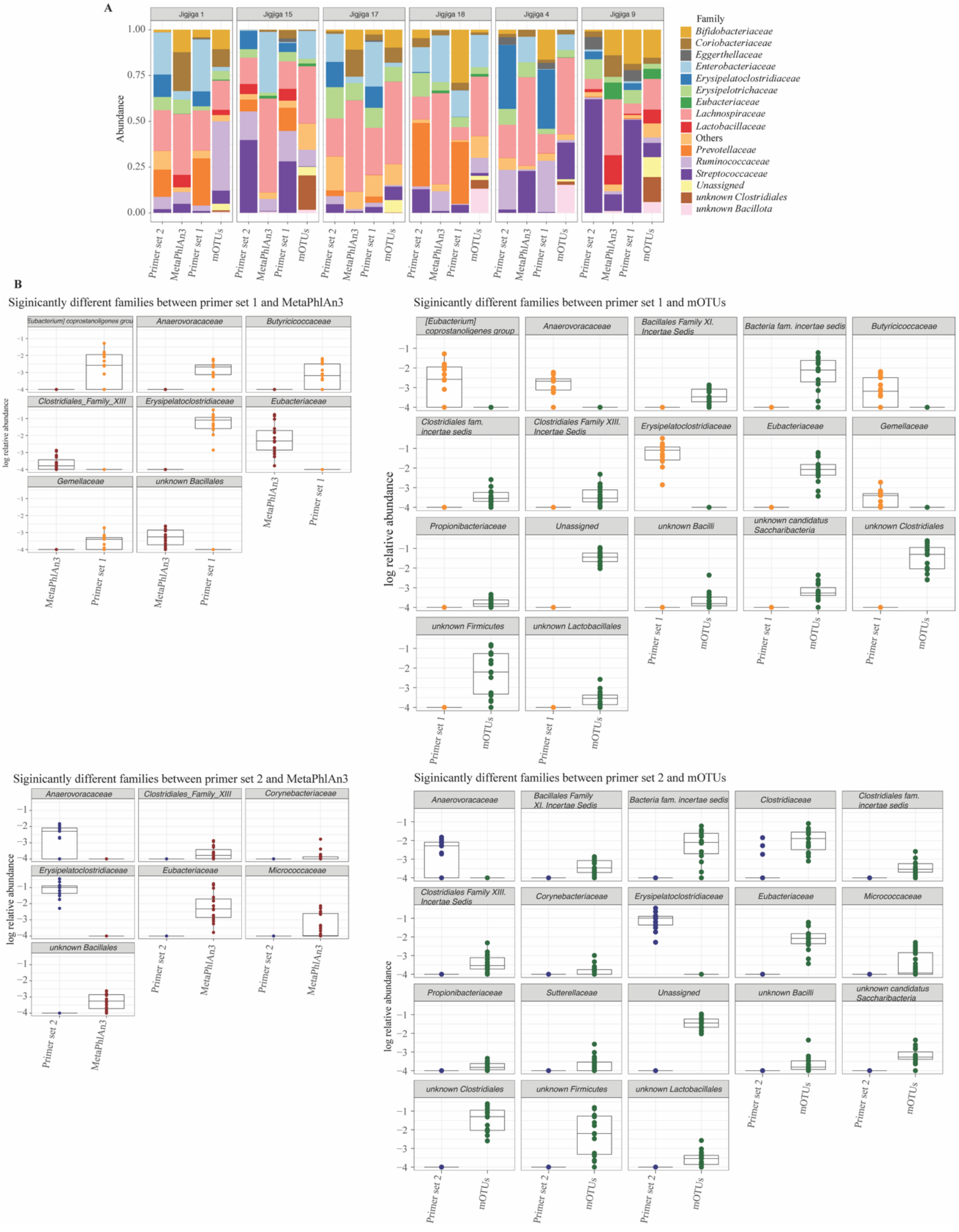

Figure S8: Comparison of the 6 samples sequenced using all strategies. *A. Comparison at the family level of the 6 samples sequenced using both primer set, as well as by shotgun metagenomic sequencing and characterize by both mOTUs2 and MetaPhlAn3. B. Families with a significantly different abundance between the two primer set and the two profiling tools, mOTUs2 and MetaPhlAn3.*

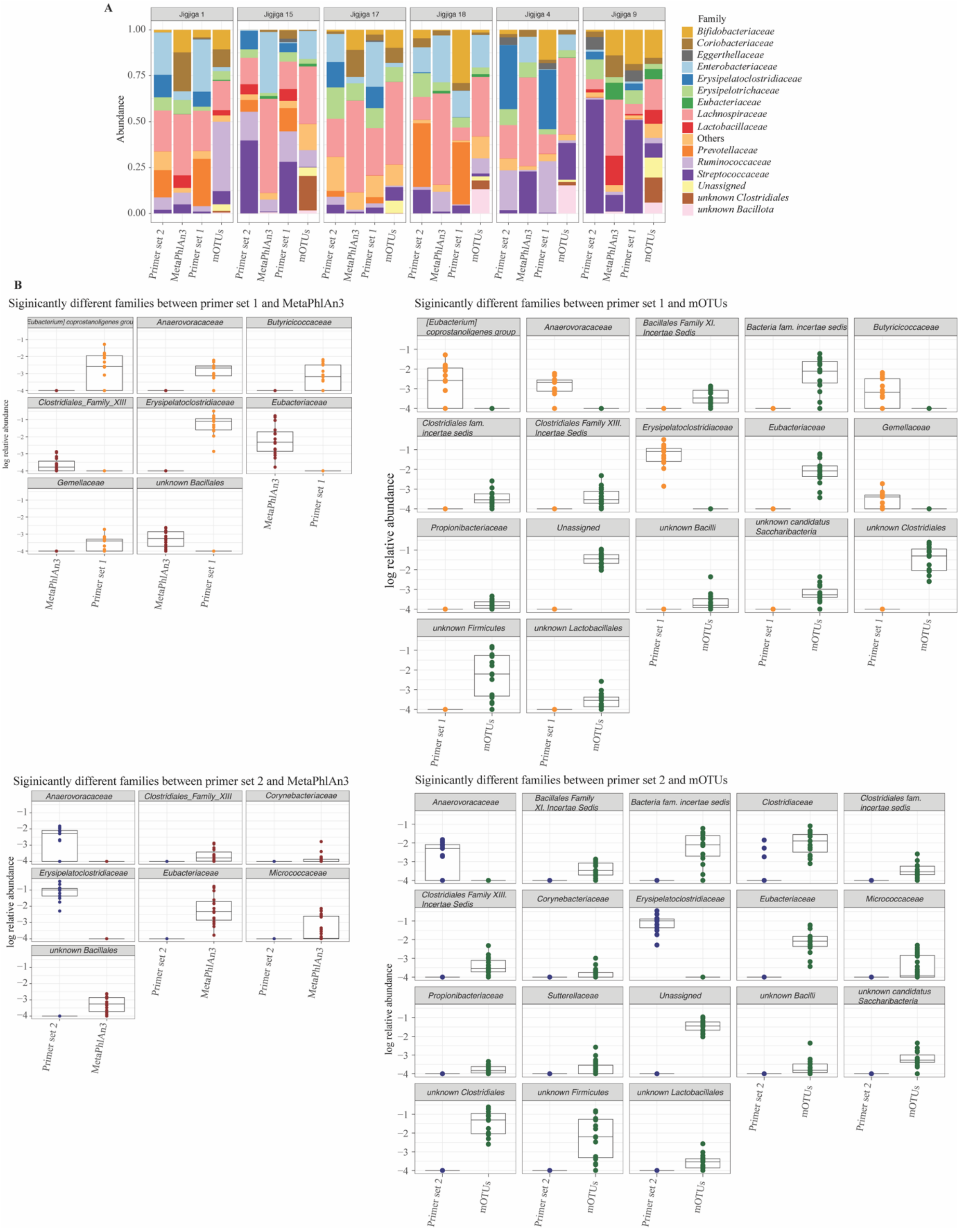

Figure S9: Taxonomic composition using mOTUs2 on shotgun metagenomic samples*. A. Top 10 most abundant families. B. VANISH/BloSSUM families (Akkermansiaceae, Bacteroidaceae, Prevotellaceae, Succinivibrionaceae), Bifidobacteriaceae, Erysipelotrichaceae, Lactobacillaceae, and Streptococcaceae relative abundance comparison between lifestyles (Wilcoxon rank test at a significance level of 0.05 with Bonferroni correction, (NS P > 0.05, * P < 0.05, ** P <*

*0.01, *** P < 0.001). C. PCoA of Bray-Curtis's distance. D. Heatmap with clustering of the samples and taxa with significantly different abundance between the two clusters (Wilcoxon rank test at 0.05 with Bonferroni correction).*

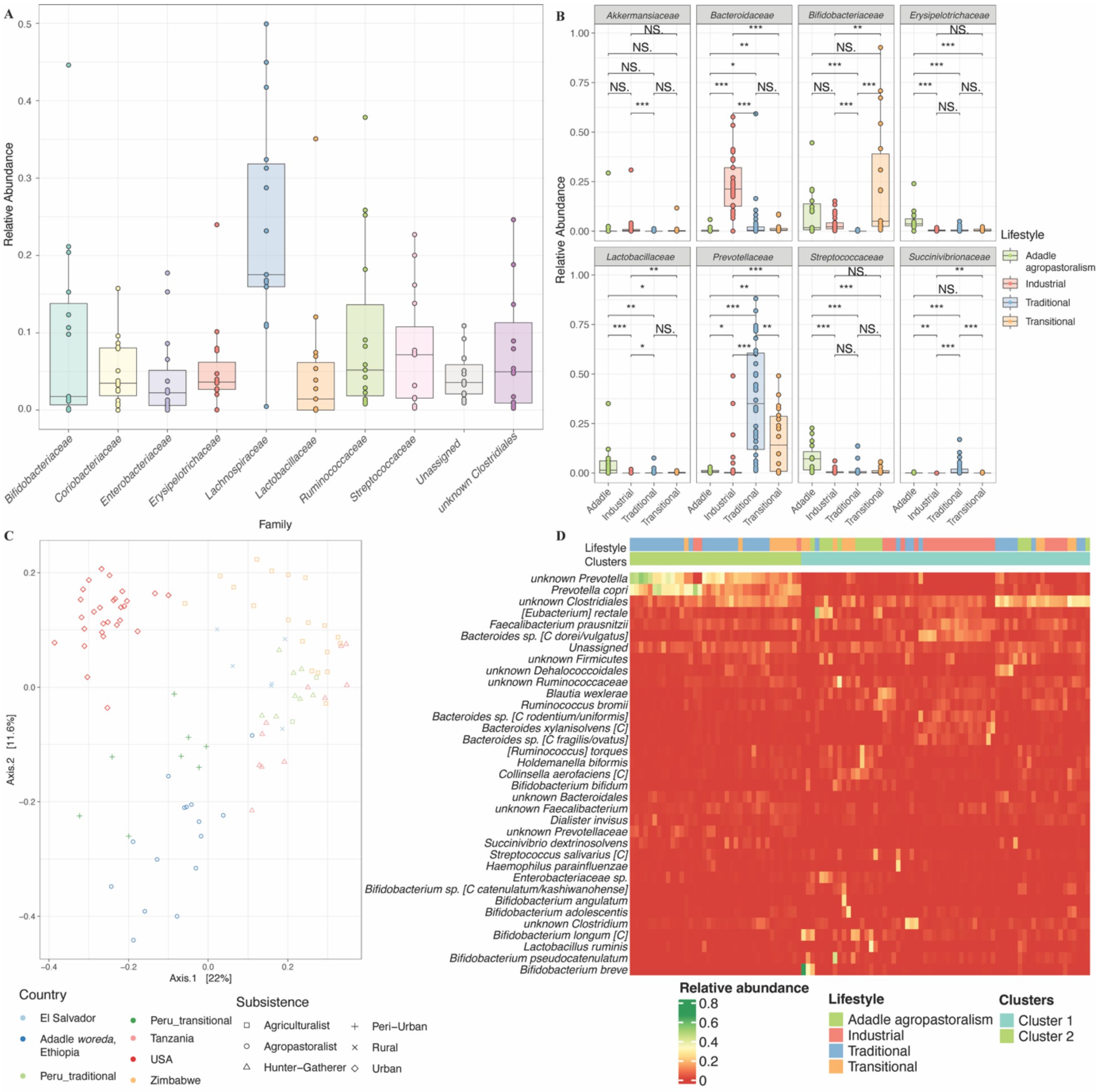

***Figure S10: MetaCyc pathways grouped at the superclass 1 and superclass 2 level**. A. Stacked barplot of the pathways at the superclass 1 level including unintegrated and unmapped pathways. B. Heatmap of the pathways at the superclass 1 level, ordered by relative abundance. C. Heatmap of the pathways at the superclass 2 level ordered by relative abundance.*

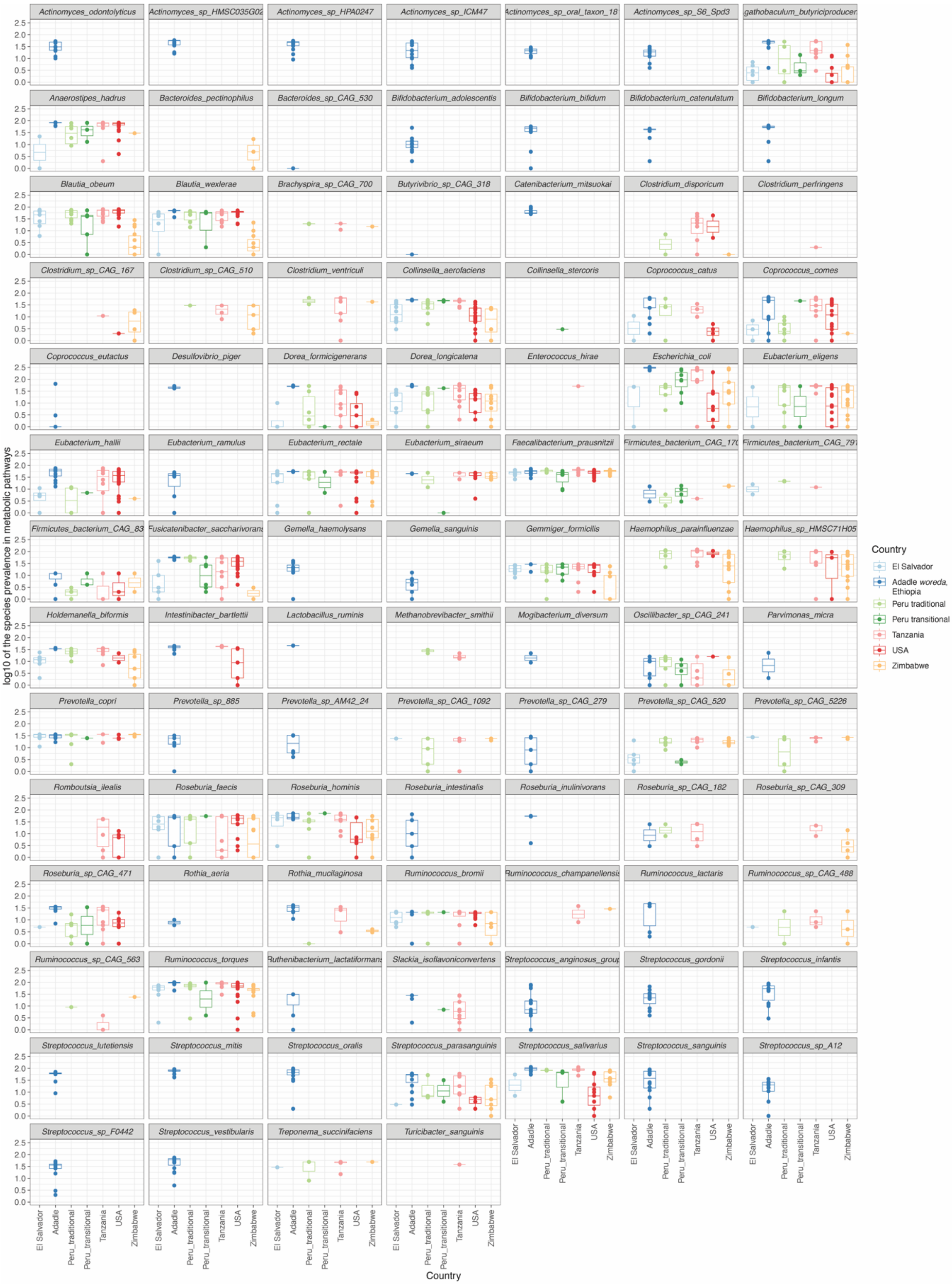

Figure S11: log10 of number of metabolic pathways in which each species contributed to in each country.

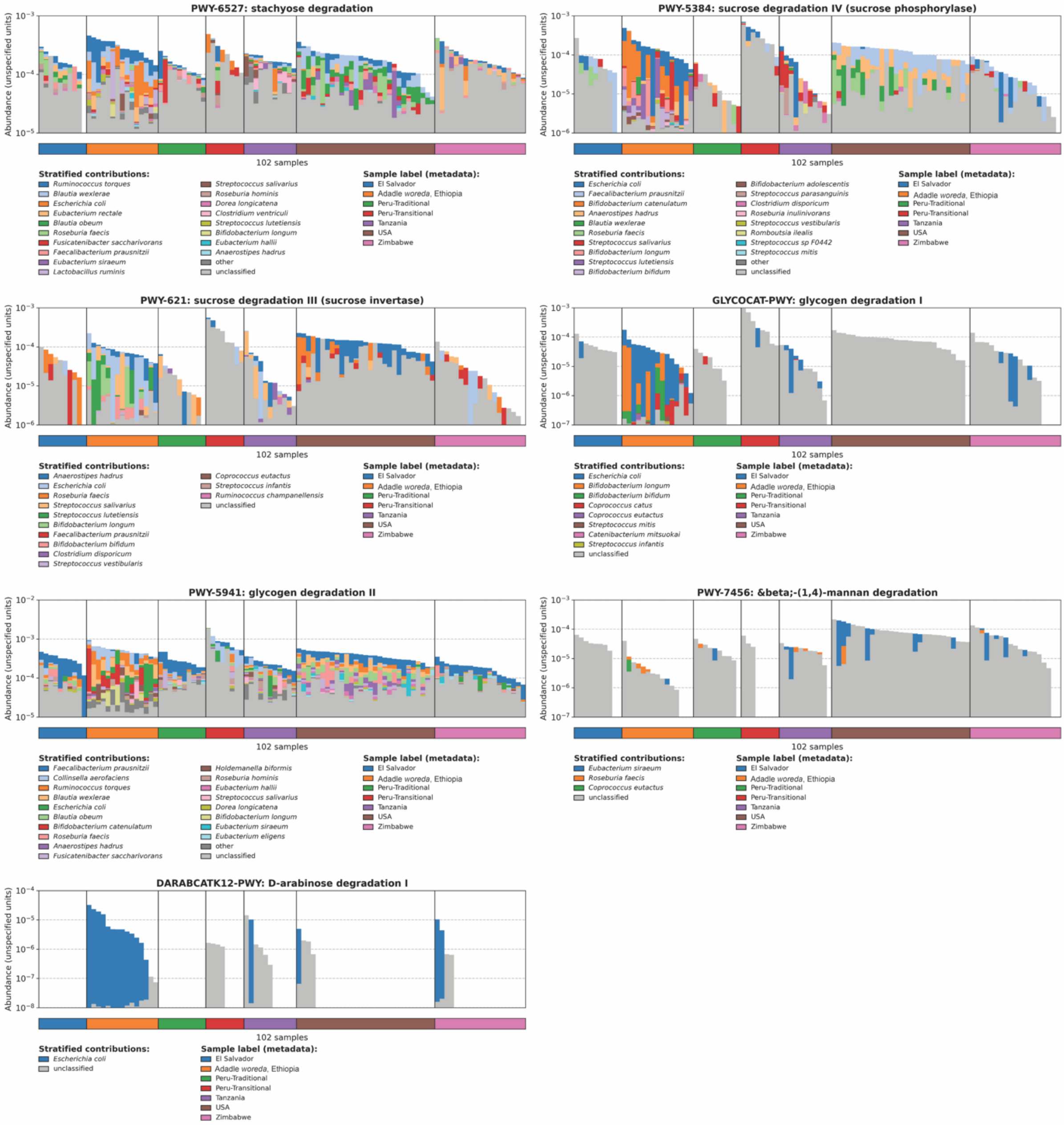

Figure S12: HUMAaN3 barplots of carbohydrate degradation pathways with species stratified contribution and ordered by country. *Sample label: country of origin of the samples.*

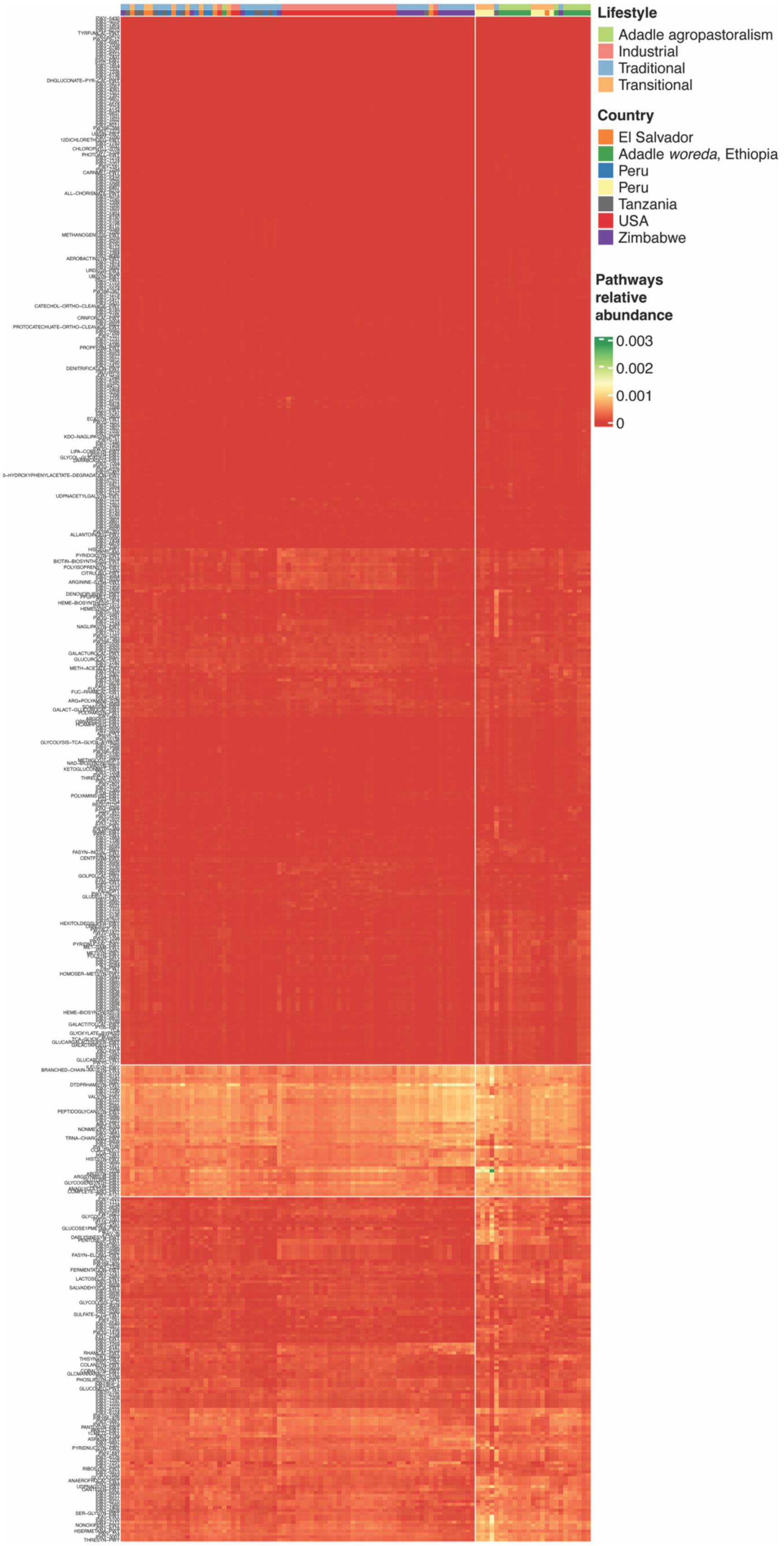

Figure S13: Heatmap of all pathways found with HUMAaN3 and clustering of the samples.

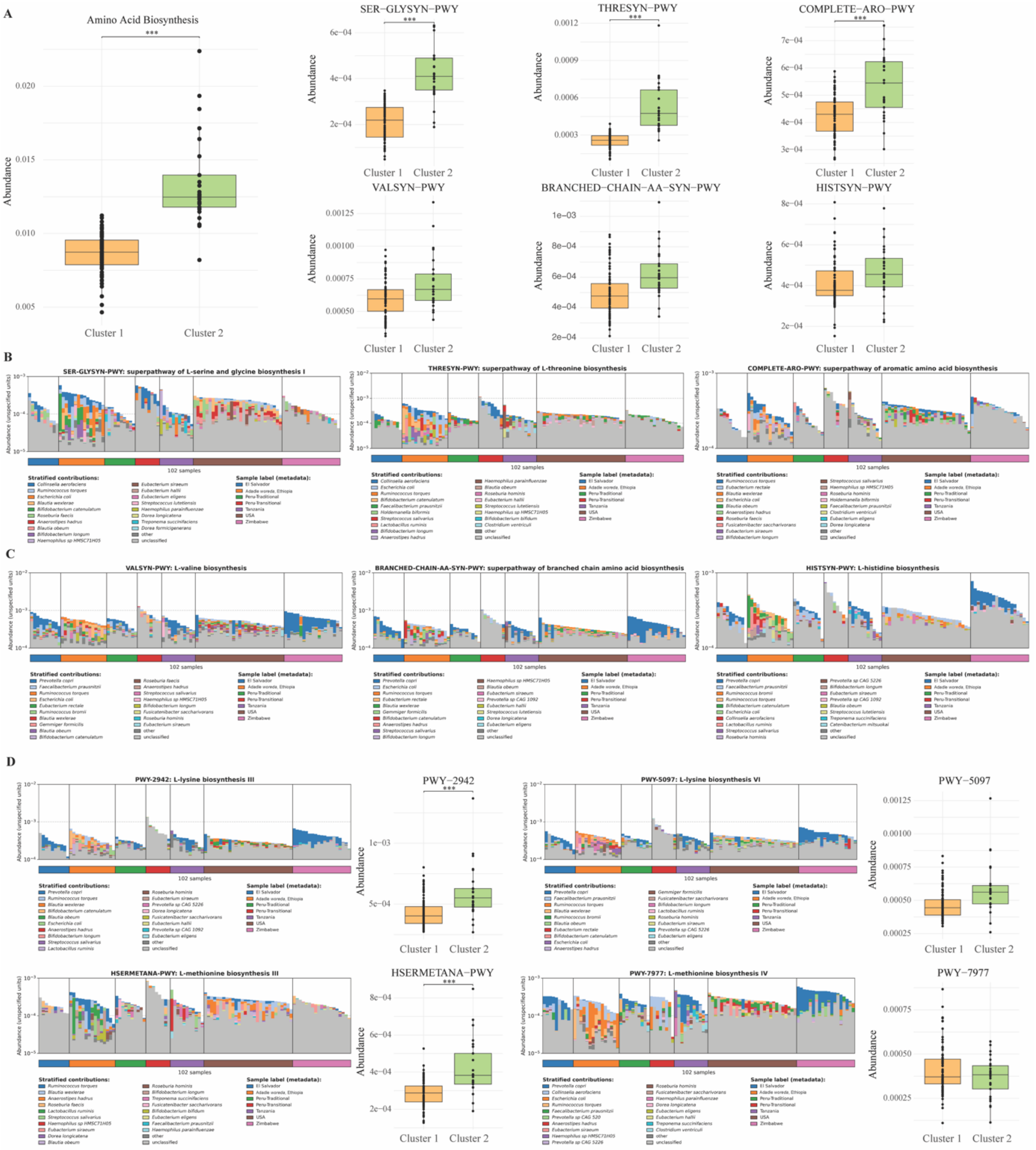

Figure S14:Amino Acid Biosynthesis pathways enrichment*: A. Abundance of superclass 2 Amino Acid Biosynthesis and enrichment of SER-GLYSYN-PWY, THRESYN-PWY, COMPLETE-ARO-PWY, VALSYN-PWY, BRANCHED-CHAIN-AA-SYN-PWY and HISTSYN-PWY between clusers 1 and 2. B. HUMAaN3 barplots of pathways SER-GLYSYN-PWY, THRESYN-PWY and COMPLETE-ARO-PWY as an example of enriched amino acid biosynthesis pathways in cluster 2. C. HUMAaN3 barplots of pathways VALSYN-PWY, BRANCHED-CHAIN-AA-SYN-PWY and HISTSYN-PWY as an example of amino acid biosynthesis pathways not enriched in any cluster. D. HUMAaN3 barplots of pathwaysPWY-2942, PWY-5097, HSERMETANA-PWY and PWY-7977 as an example of pathways with contrasting results. L-lysine biosynthesis III enriched*

in cluster 2 but L-lysine biosynthesis VI not enriched in neither of the clusters and L-methionine biosynthesis III biosynthesis enriched in cluster 2 but L-methionine biosynthesis IV not enriched in neither of the clusters.

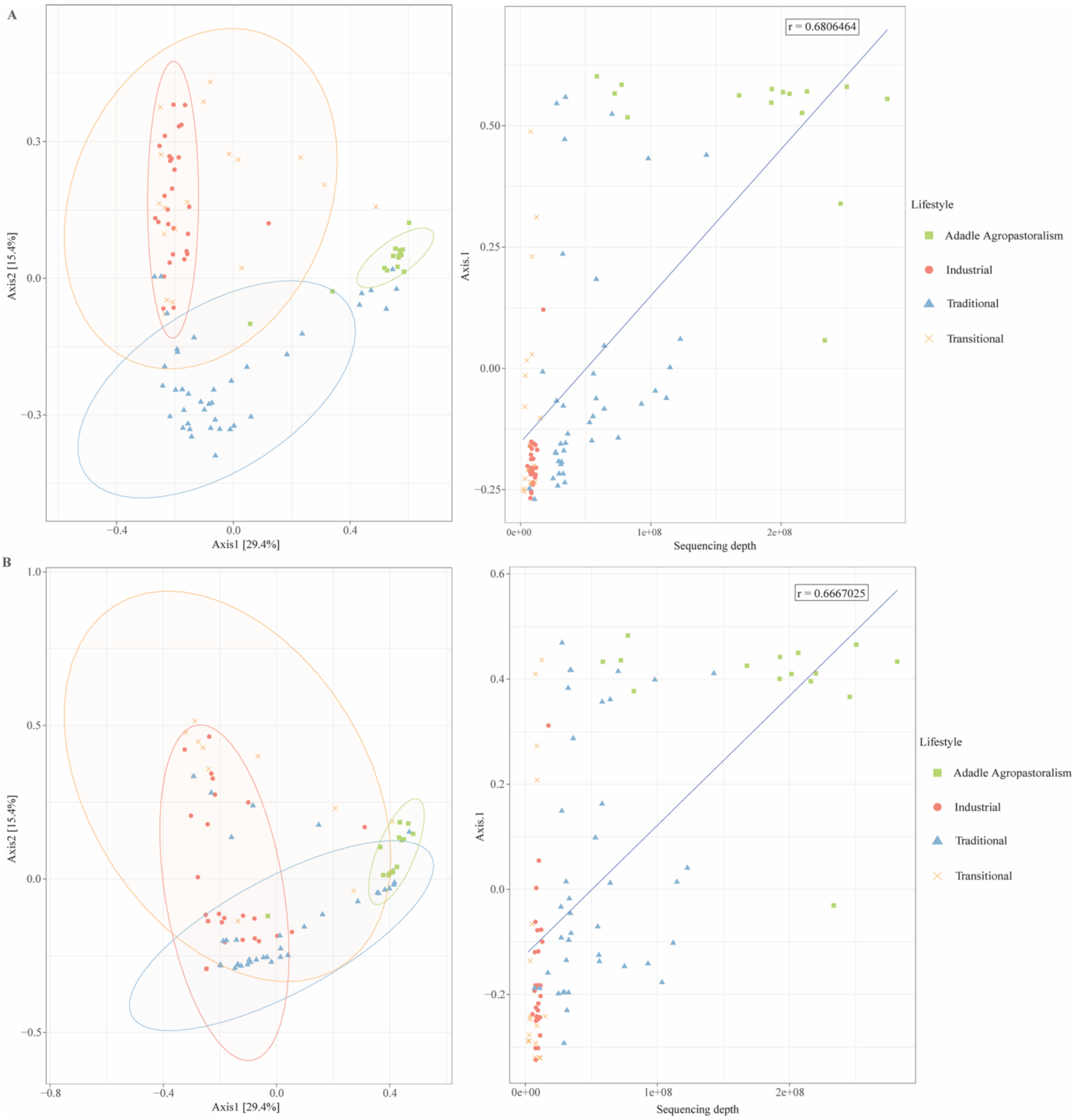

Figure S15: Presence and absence analysis of the AMR genes and drug classes. *(A) Left: PCoA of the Jaccard distance on the AMR genes. Right: Correlation plot of the sequencing depth and the position on the Axis 1 of the PCoA for the AMR genes. (B) Left: PCoA of the Jaccard distance on the drug classes. Right: Correlation plot of the sequencing depth and the position on the Axis 1 of the PCoA for the drug classes.*

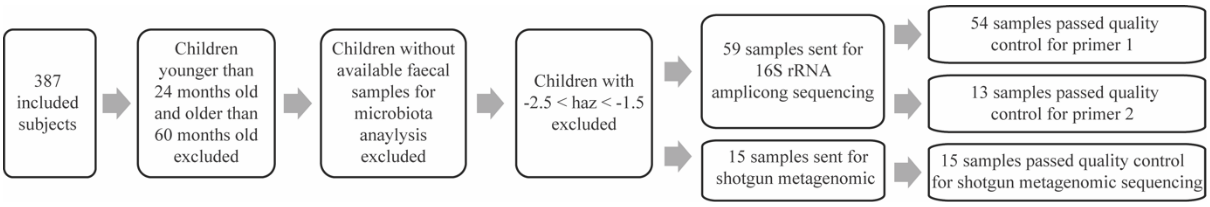

Figure S16: Inclusion criteria for the fecal samples and number of samples sent for sequencing.

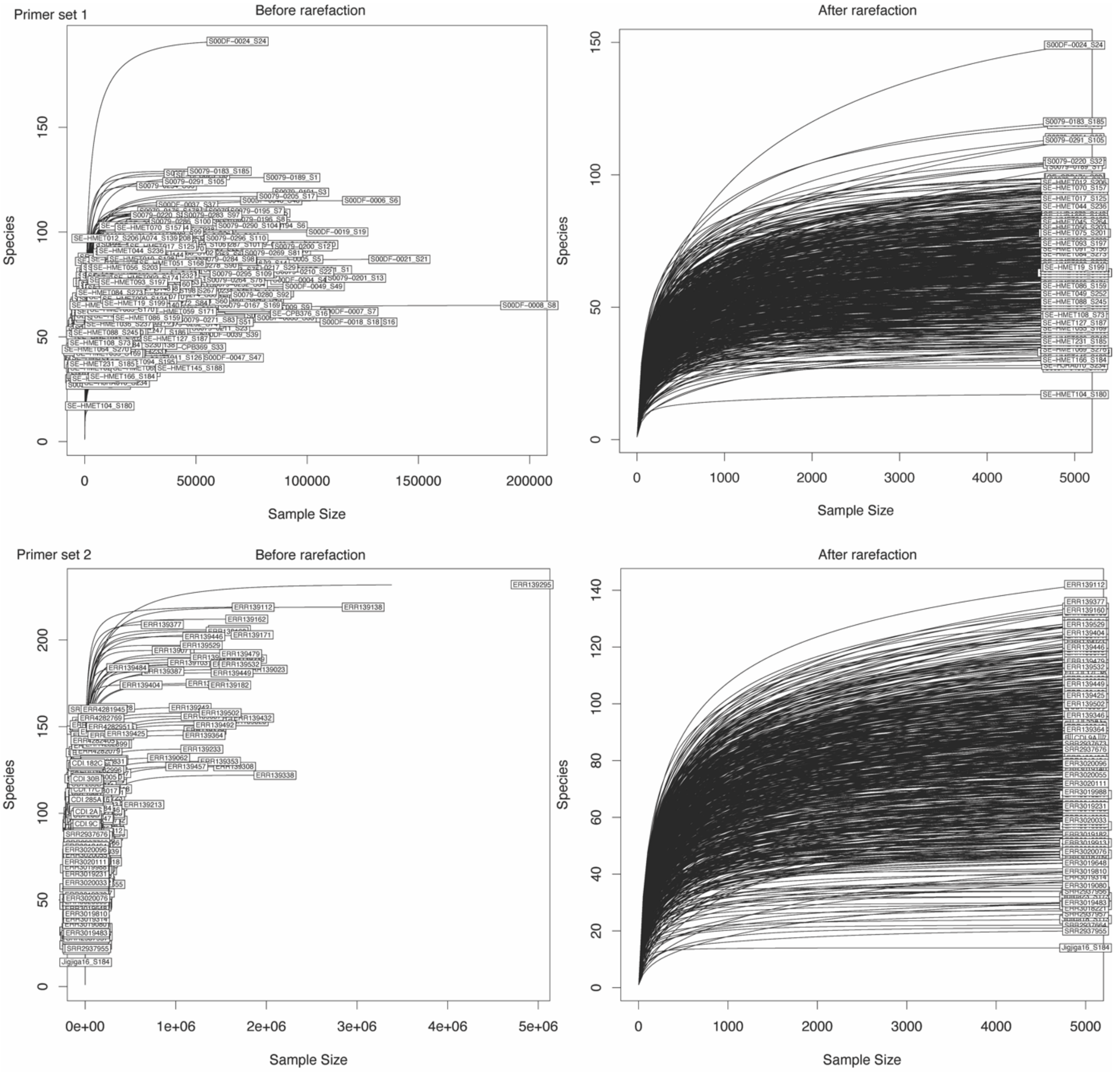

***Figure S17: Rarefaction curves**. Left before rarefaction, right after rarefaction at 5’000 reads. Primer set 1 are primers v4.SA501-v4.SA508/v4.SA701-v4.SA712. Primer set 2 are primers 515F/806R.*
